# What is next for LLMs? Pushing the boundaries of next-gen AI computing hardware with photonic chips

**DOI:** 10.1515/nanoph-2025-0217

**Published:** 2025-10-06

**Authors:** Renjie Li, Qi Xin, Wenjie Wei, Sixuan Mao, Erik Ma, Zijian Chen, Jingxing Gao, Malu Zhang, Haizhou Li, Zhaoyu Zhang

**Affiliations:** School of Science and Engineering, Guangdong Key Laboratory of Optoelectronic Materials and Chips, Shenzhen Key Lab of Semiconductor Lasers, The Chinese University of Hong Kong, Shenzhen, China; University of Illinois Urbana-Champaign, Champaign, USA; University of Electronic Science and Technology of China, Chengdu, China; University of California, Berkeley, Berkeley, USA; School of Data Science, The Chinese University of Hong Kong, Shenzhen, China; National University of Singapore, Singapore, Singapore

**Keywords:** LLM, photonics, artificial intelligence, spintronics, spiking neural network, neuromorphic computing

## Abstract

Large language models (LLMs) are rapidly pushing the limits of contemporary computing hardware. For example, training GPT-3 has been estimated to consume around 1,300 MWh of electricity, and projections suggest future models may require city-scale (gigawatt) power budgets. These demands motivate exploration of computing paradigms beyond conventional von Neumann architectures. This review surveys emerging photonic hardware optimized for next-generation generative AI computing. We discuss integrated photonic neural network architectures (e.g. Mach–Zehnder interferometer meshes, lasers, wavelength-multiplexed microring-resonators) that perform ultrafast matrix operations. We also examine promising alternative neuromorphic devices and platforms, including 2D materials and hybrid spintronic–photonic synapses, which combine memory and processing. The integration of two-dimensional materials (graphene, TMDCs) into silicon photonic platforms is reviewed for tunable modulators and on-chip synaptic elements. Transformer-based LLM architectures (self-attention and feed-forward layers) are analyzed in this context, introducing the mathematical operations associated with the transformers and identifying strategies and challenges for mapping dynamic matrix multiplications onto these novel photonic hardware systems. Overall, we broadly introduce state-of-the-art photonic components, AI algorithms, and system integration methods, highlighting key advances and open issues in scaling such photonic systems to mega-sized LLM models. We find that photonic computing systems could potentially surpass electronic processors by orders of magnitude in throughput and energy efficiency, but require breakthroughs in memory especially for long-context windows and long token sequences and in storage of ultra-large datasets, among others. This survey provides a comprehensive roadmap for AI hardware development, emphasizing the role of cutting-edge photonic components and technologies in supporting future LLMs.

## 1 Introduction

The recent proliferation of transformer-based large language models (LLMs) has dramatically increased the demands on computing infrastructure. Training state-of-the-art AI models now requires enormous compute and energy resources. For example, the GPT-3 model consumed an estimated 1.3 × 10^3^ MWh of electricity during training, and industry projections suggest that next-generation LLMs may demand power budgets on the order of gigawatts. These trends coincide with the use of massive GPU clusters (for instance, Meta has trained Llama 4 on a cluster exceeding 10^5^ NVIDIA H100 GPUs). Meanwhile, conventional silicon scaling is approaching fundamental limits (transistors are reaching ∼3 nm feature sizes), and von Neumann architectures suffer from memory-processor bottlenecks that constrain speed and energy efficiency [1]. Together, these factors underscore a growing gap between the computational demands of LLMs and the capabilities of traditional CMOS electronic hardware [1]. These challenges have spurred exploration of alternative computing paradigms. Photonic computing, which processes information with light, offers intrinsic high bandwidth, massive parallelism, and minimal heat dissipation [1]. Recent advances in photonic integrated circuits (PICs) have enabled neural-network primitives such as coherent interferometer meshes, microring-resonator (MRR) weight banks, and wavelength-division multiplexing (WDM) schemes to perform dense matrix multiplications and multiply-accumulate operations at the speed of light. Such photonic processors exploit WDM to achieve extreme parallelism and throughput. Simultaneously, integrating two-dimensional (2D) materials (graphene, TMDCs) into PICs has produced ultrafast electro-absorption modulators and saturable absorbers that serve as on-chip neurons and synapses. Complementary to optics, spintronic neuromorphic devices (e.g., magnetic tunnel junctions and skyrmion channels) offer non-volatile synaptic memory and spiking neuron behavior. These photonic and spintronic neuromorphic elements inherently co-locate memory and processing and leverage new physical mechanisms for energy-efficient AI computation. Mapping transformer-based LLM architectures onto these emerging hardware substrates raises unique challenges. Transformer self-attention layers involve dynamically computed weight matrices (queries, keys, and values) that depend on the input data. Designing reconfigurable photonic or spintronic circuits to realize such data-dependent operations is an active area of research. Furthermore, implementing analog nonlinearities (e.g. GeLU activation) and normalization in optical/spintronic media remains a major challenge. Addressing these issues has motivated hardware-aware algorithm design, such as photonics-friendly training methods and neural network models that tolerate analog noise and quantization.

The pursuit of neuromorphic computing stems from fundamental limitations in conventional von Neumann architectures. Traditional computing systems suffer from the “von Neumann bottleneck” [2], where physical separation between processing and memory units leads to excessive energy consumption and latency during data transfer. This bottleneck is exacerbated by the growing performance gap between processors and memory, known as the “memory wall” [3]. Modern computers require megawatts of power to simulate basic brain functions [4], while biological brains achieve remarkable cognitive capabilities with merely 20 W [5]. Simultaneously, the semiconductor industry faces existential challenges as transistor miniaturization approaches physical limits and Moore’s law stagnates [6], [7], [8]. These dual crises in architecture and transistor scaling have driven intense interest in brain-inspired computing paradigms. Neuromorphic computing addresses these challenges through three key innovations: 1) co-location of computation and memory, 2) analog information encoding, and 3) massively parallel connectivity [9], [10], [11], [12], [13], [14]. While theoretical frameworks for neural networks date back to McCulloch and Pitts’ binary neuron model (1943) and subsequent developments in deep learning [15], [16], practical implementations face significant hardware constraints. CMOS-based implementations using transistor arrays [17] lack essential neurobiological features like nonlinear dynamics, long-term plasticity, and stochasticity [9]. The emergence of nonvolatile memory technologies – particularly memristors [18], [19] – has enabled more biologically plausible implementations, but material limitations persist. Resistive RAM (RRAM) [20], [21], [22], [23], [24], [25], phase-change materials [26], [27], [28], [29], [30], and ferroelectric devices [31], [32], [33], [34], [35] face tradeoffs between endurance, speed, and controllability that constrain large-scale deployment.

**Figure 1:**
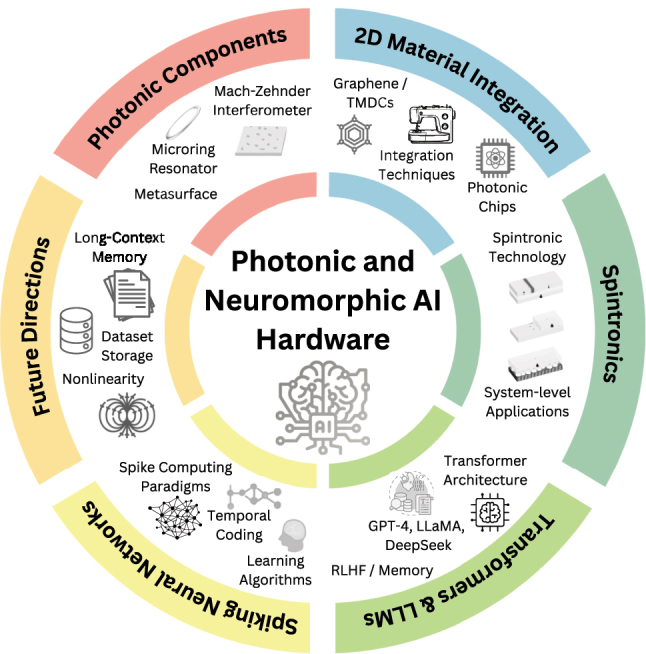
Overall organization of this review article.

As shown in Figure 1, the remainder of this review is organized in the following manner. Section 2 surveys photonic accelerator architectures, including coherent interferometer meshes, microring weight banks, and WDM-based matrix processors. Section 3 discusses the integration of two-dimensional materials into photonic chips (graphene/TMDC modulators, photonic memristors). Section 4 examines alternative neuromorphic devices, covering optical spintronics for neuromorphic computing. Section 5 summarizes the principles of mainstream LLMs and transformers and how they can be mapped onto photonic chips, highlighting strategies for implementing attention and feed-forward layers in photonic and neuromorphic hardware. Finally, Section 6 identifies key system-level challenges and outlines future directions. Through this comprehensive survey, we aim to chart a roadmap for next-generation AI hardware development using photonic technologies.

## 2 State-of-the-art photonic components for photonic neural networks and photonic computing

Existing literature presents distinct classification schemes for Photonic Neural Networks (PNNs) [36], [37], [38], [39]. For instance, Liao et al. [36] categorize Integrated Photonic Neural Networks (IPNNs) by their underlying photonic architecture, dividing them into waveguide-based, dielectric metasurface-based, and photonic spiking neural networks. In contrast, De Marinis et al. [37] classify PNNs based on their neural network architectures, such as multilayer perceptrons (MLPs) and convolutional neural networks (CNNs). Building on these diverse approaches, this work introduces PNNs based on optical components.

Photonic neural networks (PNNs) are increasingly demonstrating capabilities surpassing electronic devices. For example, Chen et al. [40] developed a VCSEL-ONN system that can reach a computational density of 6 tera OP mm^−2^s^−1^ – 20 times higher than its electronic counterparts. PNNs leverage the synergistic effects of various optical components to achieve efficient computation: microring resonators utilize resonance effects for wavelength multiplexing and optical frequency comb generation, providing the foundation for multi-wavelength signal processing [41], [42], [43]; Mach–Zehnder interferometer (MZI) arrays perform optical matrix operations through phase modulation, enabling core linear transformations in neural networks [44], [45], [46], [47]; metasurfaces manipulate the phase and amplitude of light waves via subwavelength structures, executing highly parallel optical computations in the diffraction domain [48], [49], [50], [51], [52], [53], [54], [55], [56]; the 4f system performs linear filtering in the diffraction domain through Fourier transform [57], [58]; while novel lasers achieve nonlinear activation through electro-optic conversion via diverse approaches [40], [59], [60], [61]. By integrating optical field manipulation, linear transformations, and nonlinear responses, these components construct all-optical computing architectures with high speed, low power consumption, and massive parallelism. This section introduces the optical devices commonly employed in current optical neural network (ONN) implementations.

### 2.1 Microring resonator

**Figure 2:**
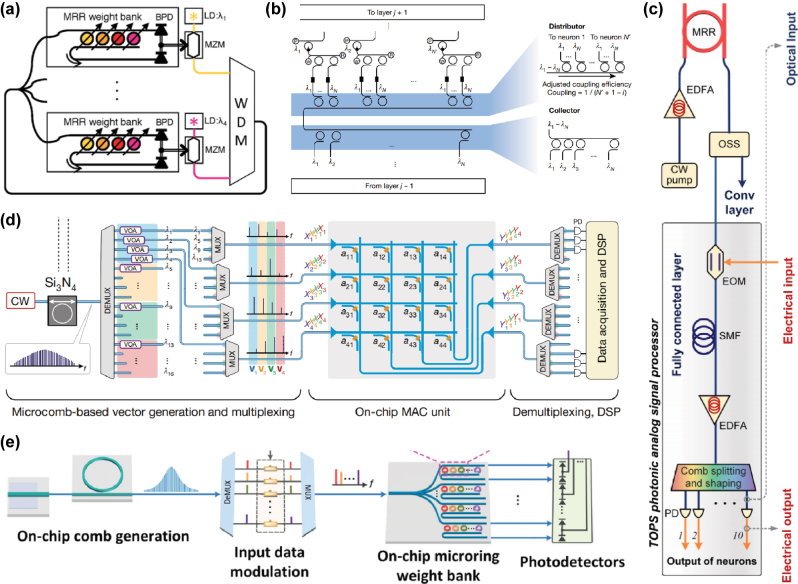
Microring resonator: (a) Neuromorphic ONNs can be realized through microring resonator (MRR) weight banks [62]. (b) The underlying mechanism and experimental setup of fully optical spiking neural networks are illustrated in [26]. (c) A photonic convolution accelerator has been developed using a time-wavelength multiplexing approach [41]. (d) In-memory photonic computing architectures leverage on-chip microcombs and phase-change materials [42]. (e) Microcomb-based integrated ONNs enable convolution operations for applications such as emotion recognition. [43]. (a) is reprinted from Ref. [62], with permission. Copyright 2024 Nature; (b) is reprinted from Ref. [26], with permission. Copyright 2024 Nature; (c) is reprinted from Ref. [41], with permission. Copyright 2024 Nature; (d) is reprinted from Ref. [42], with permission. Copyright 2024 Nature; (e) is reprinted from Ref. [43], with permission (CC BY 4.0).

The significance of microring resonators (MRRs) (Figure 2) extends beyond their role as waveguides for wavelength-division multiplexing (WDM) [41], [42], [43] to their unique filtering capabilities, such as optical frequency comb generation [41], [42], [43]. On the one hand, WDM allows simultaneous propagation of different wavelength signals in the same structure without inter-channel interference: By designing the radius and refractive index of MRRs to support specific resonant wavelengths, light matching the resonance condition becomes coupled into the ring cavity for sustained oscillation, manifesting as distinct absorption dips in the transmission spectrum. On the other hand, the optical frequency combs arise from parametric oscillations in high-Q (low-loss) microresonators: When a continuous-wave (CW) pump laser is injected, photons experience nonlinear effects (e.g., Kerr nonlinearity), spontaneously generating equidistant spectral lines that form a comb-like spectrum. The interplay between WDM and comb generation allows multi-wavelength signals to be simultaneously synthesized and transmitted through shared waveguides, achieving both wavelength multiplexing and spatial multiplexing capabilities.

Other properties of microrings have also been exploited. For example, paper [26] utilized the thermo-optic effect of microrings paper [26], [42] installed phase-change materials with a lasing threshold on the ring to achieve a nonlinear effect similar to the ReLU function in neural networks.

### 2.2 Mach–Zehnder interferometer

**Figure 3:**
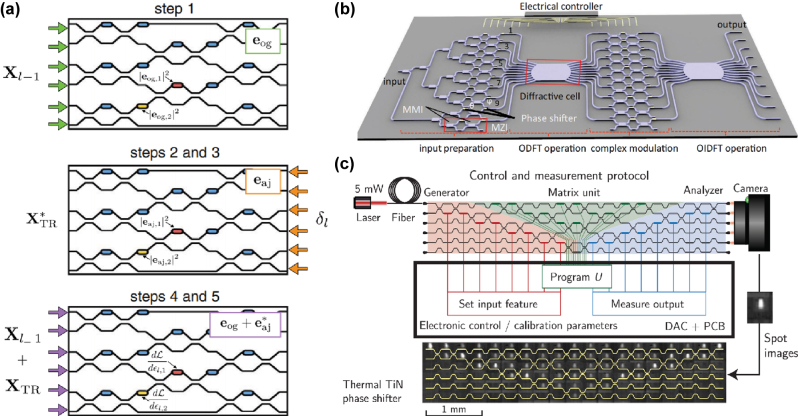
Mach–Zehnder Interferometer: (a) Training methodology diagram for ONNs enabling real-time *in-situ* learning [45]. (b) Integrated photonic neural network architecture combining MZIs with diffractive optical components [46]. (c) Demonstrated *in situ* backpropagation training of a photonic neural network using MZI meshes. [47]. (a) is reprinted from Ref. [45], under the terms of the Open Access Publishing Agreement; (b) is reprinted from Ref. [46], with permission. Copyright 2024 Nature; (c) is reprinted from Ref. [47], with permission. Copyright 2024 American Association for the Advancement of Science.

MZI arrays (Figure 3) can effectively performing optical matrix-vector multiplication (MVM) [44], [45], [46], [47]: It is composed of two optical couplers/splitters and two modulators (which can be controlled via external circuits). The input light is split into two arms by the splitter, and the phase difference between them is adjusted by the modulators. Finally, the light is recombined through the optical coupler, resulting in interference. Each MZI performs a 2D unitary transformation (orthogonal transformation in the complex domain) on optical signals, mathematically equivalent to a 2 × 2 unitary matrix. When multiple MZIs are cascaded in specific topologies (e.g., mesh configurations), their collective behavior corresponds to the decomposition of a high-dimensional unitary matrix since any N-dimensional unitary matrix can be decomposed into a sequence of 2D unitary operations. Thus MZI arrays can implement programmable unitary transformations analogous to weight matrices in neural networks.

The output optical signals can be further converted through optoelectronic means and integrated with electronic devices to implement nonlinear activation functions, completing the forward propagation of the neural network. It should be noted that the network depicted in Figure 3b not only employs MZIs but also utilizes diffractive optical elements to perform Fourier and inverse Fourier transforms.

### 2.3 Metasurface

The operation of metasurfaces in neural network applications primarily relies on the diffraction and interference of light between “surfaces” [48], [49], [50], [51], [52], [53], [54], [55], [56]. A metasurface is a material composed of subwavelength-scale structural elements that can modulate optical wave properties including phase, amplitude, polarization, and frequency. These structures typically exhibit ultra-thin profiles, lightweight characteristics, and high integration density (with massive parallelism), with diverse implementations such as silicon-on-insulator (SOI)-based designs [49], [51], compound Huygens’ metasurfaces [50], and single-layer holographic perceptrons [52]. Since diffraction and interference are inherently linear processes, achieving nonlinear computation requires additional mechanisms, such as leveraging the optoelectronic effects of metasurface materials [53].

**Figure 4:**
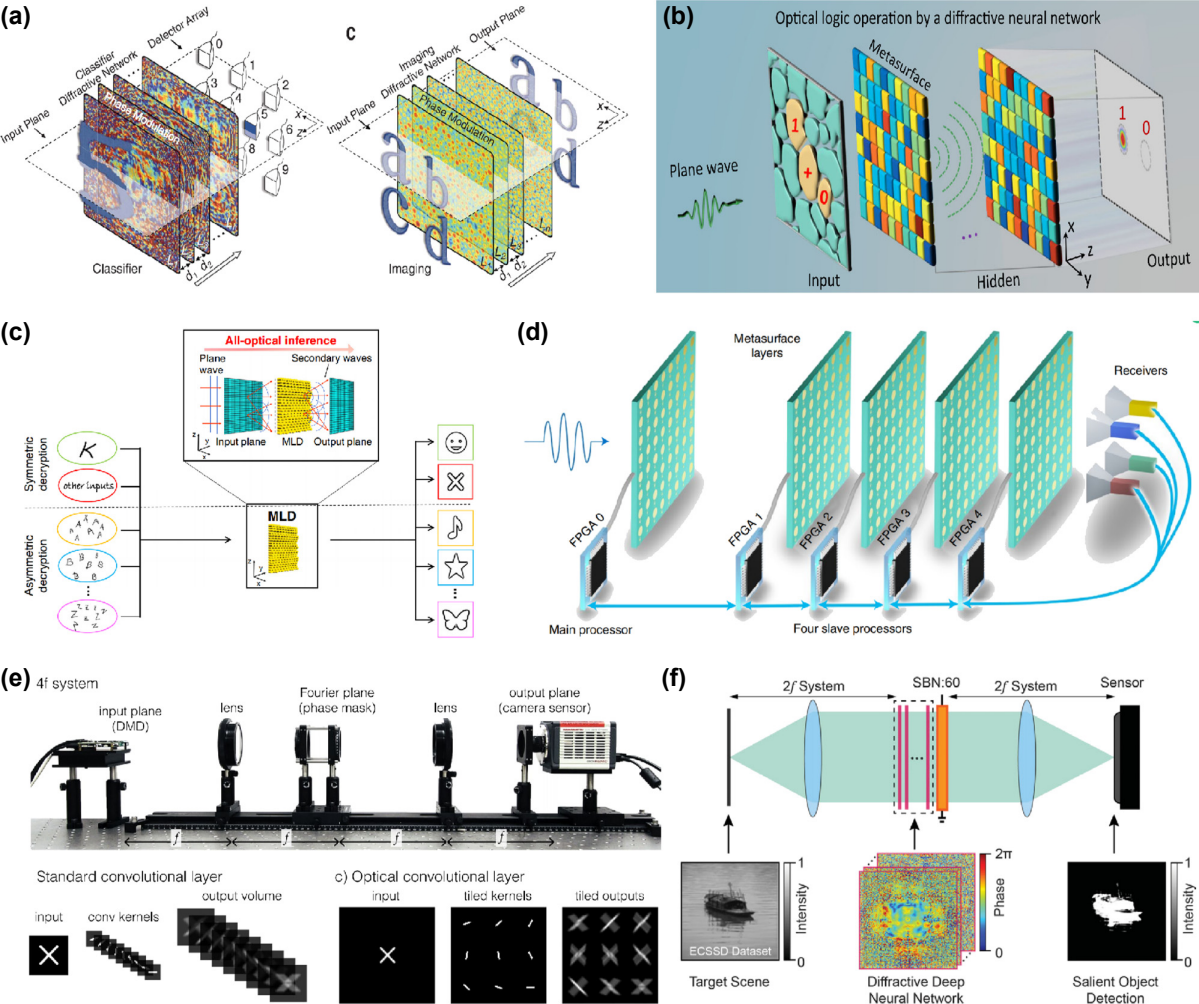
Free-space PNNs using Metasurface: (a) Conceptual representation of the inference mechanism in diffractive deep neural networks (D2NN) [48]. (b) Experimental configuration demonstrating logical operations through diffractive optical neural networks (DONN) [50]. (c) Nanoprinted optical perceptrons enable on-chip [52]. (d) Reconfigurable DONN architecture utilizing digital meta-atom arrays [55]. (e) A hybrid optoelectronic CNN using 4f optical setup [58]. (f) An entirely ONN architecture where a deep diffractive neural network is integrated into the Fourier plane of a 4f imaging system. [57]. (a) is reprinted from Ref. [48], with permission. Copyright 2024 American Association for the Advancement of Science; (b) is reprinted from Ref. [50], with permission. Copyright 2024 Nature; (c) is reprinted from Ref. [52], with permission. Copyright 2024 Nature; (d) is reprinted from Ref. [55], with permission. Copyright 2024 Nature; (e) is reprinted from Ref. [58], with permission. Copyright 2024 Nature; (f) is reprinted from Ref. [57], with permission. Copyright 2024 American Physical Society.

**Figure 5:**
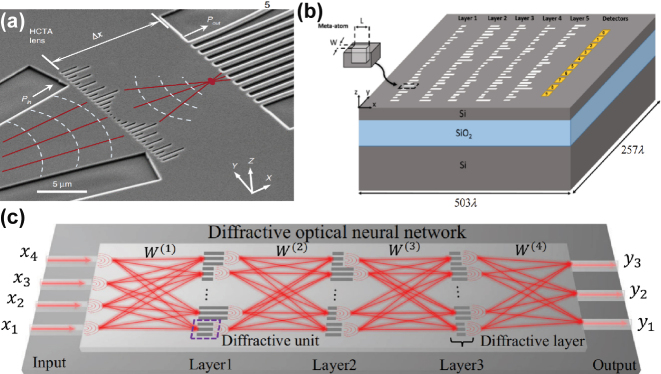
On-chip PNNs using Metasurface: (a) Experimental validation of 1D DONNs for photonic machine learning [49]. (b) Simulation-based validation of on-chip DONN with light-speed computation [51]. (c) Dielectric metasurface enables on-chip wavefront control for Fourier transform and spatial differentiation. [56]. (a) is reprinted from Ref. [49], with permission. Copyright 2024 Nature; (b) is reprinted from Ref. [51], under the terms of the Open Access Publishing Agreement; (c) is reprinted from Ref. [56], with permission. Copyright 2024 Nature.

Multilayer diffractive architectures (Figure 4) [48], [50], [53], [54], [55] employ stacked 2D surfaces as densely arranged neuron layers. Through controlled modulation of relative thickness or material properties at each spatial position in the diffraction layers, phase and amplitude adjustments of light are achieved. To enable such structures, certain researchers utilize 4f optical systems [57], [58]. The 4f system employs optical field signals (e.g., images) that undergo Fourier transformation through the first lens. At the Fourier plane behind the lens, metasurfaces perform spectral filtering or weight adjustment. The modulated spectrum is then inversely Fourier-transformed by a second lens to generate the output optical field. Alternatively [49], [51], [56], fabricate 1D high-contrast transmit array metasurfaces (Figure 5) on one planar surface. For example, etching air grooves (potentially later filled with silica) on standard SOI substrates, featuring fixed groove spacing (lattice constants) and width. Phase control is achieved by modulating groove length.

### 2.4 Lasers

**Figure 6:**
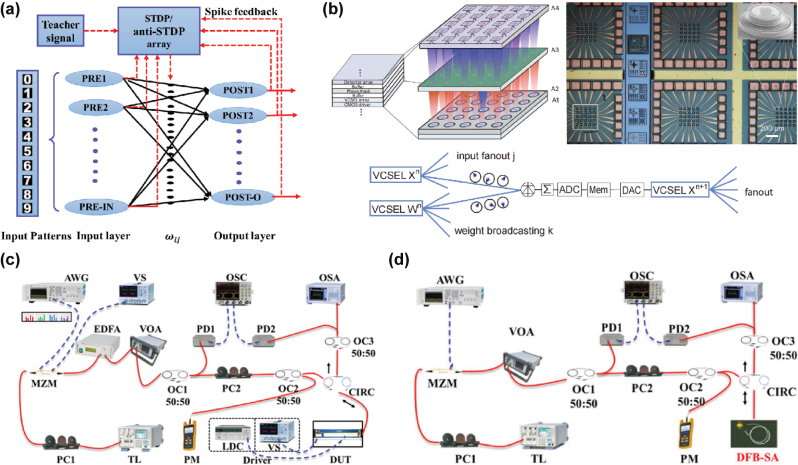
Lasers: (a) Theoretical analysis of the all-optical SNN using VCSELs [59]. (b) VCSEL-based all-optical SNN for supervised learning [40]. (c) FP-SA neuron chip for hardware-algorithm collaborative computing in SNN [61]. (d) Experimental demonstration of a photonic integrated spiking neuron using a DFB-SA laser [60]. (a) is reprinted from Ref. [59], with permission. Copyright 2024 IEEE; (b) is reprinted from Ref. [40], with permission. Copyright 2024 Nature; (c) is reprinted from Ref. [61], under the terms of the Open Access Publishing Agreement; (d) is reprinted from Ref. [60], under the terms of the Open Access Publishing Agreement.

Lasers, as a unique light source characterized by high coherence, monochromaticity, and directionality, are also utilized in ONNs, especially in spiking photonic neural networks (Figure 6).

For instance, vertical-cavity surface-emitting lasers (VCSELs) have been theoretically proposed and experimentally demonstrated in studies [40], [59]. In a VCSEL, current is injected through the electrodes into the active region, where electrons and holes recombine in the quantum well layers, emitting photons. These photons are reflected back and forth between two distributed Bragg reflector (DBR) mirrors, passing through the active region repeatedly and being amplified. When the gain (light amplification capability) exceeds the cavity losses (absorption, scattering, etc.), the threshold condition is met, and laser output is achieved [40]. One study leveraged the property of VCSEL arrays, which can maintain the same initial phase when mode-locked by a Leader Laser. In this work, feature data was encoded into electrical signals to modulate the pump voltage of one VCSEL, thereby adjusting its output light phase. Similarly, each column of the weight matrix was encoded into electrical signals to adjust the output light phases of other VCSELs. Beam splitters and couplers were used to allow the output light from the VCSEL corresponding to MNIST data to interfere with the output light from other VCSELs. Photodetectors collected the optical signals, which were summed into electrical signals as the input for the next layer of the VCSEL array, enabling forward propagation. In the final layer, the photodetector with the strongest output electrical signal corresponded to the output label.

Another example is the distributed feedback (DFB) laser with an intracavity saturable absorber (SA), referred to as DFB-SA [60]. The DFB laser’s cavity incorporates a periodic grating structure, providing optical feedback to achieve single-wavelength output. The saturable absorber (SA) region is located near the high-reflectivity end of the laser cavity. At low pump levels, the SA absorbs photons, suppressing laser output; at high pump levels, the SA allows the release of optical pulses (Q-switching effect). Therefore, when the gain current exceeds the self-pulsation threshold of the DFB-SA, the periodic absorption modulation of the SA results in pulsating output, and the output frequency exhibits a nonlinear positive correlation with the pump intensity, which can serve as the fundamental unit of a spiking neural network (SNN). Here, the DFB laser can also be replaced by a traditional Fabry–Perot (FP) laser [61].

### 2.5 Comparison with electronic chips

In this section, we incorporate more specific quantitative Key Performance Indicators (e.g., *TOPS*/*J*, *TOP*/*mm* ^2^/*s* etc.) from cited literature for specific photonic computing architectures and show direct comparisons with their electronic counterparts (e.g., Nvidia and Google’s ASICs). See this detailed comparison in Table 1. From this comparison, we see that the most advanced photonic systems already surpassed electronic systems in terms of energy efficiency and compute density by orders of magnitude, apart from latency which isn’t shown here.

**Table 1:** Key performance indicators of landmark photonic neuromorphic chips for AI and deep learning applications and their comparison to electronic counterparts.

Name abbrv.	Technologies & methods	Energy efficiency (*TOP*/*J*)	Compute density (*TOP*/*mm* ^2^/*s*)	Est. Cost /*mm* ^2^	Input encoding	Implement platform
Nvidia GPU [40]	ASIC	0.63	0.16	$	Electrical signal (binary)	CMOS Electronics
Google TPU [40]	ASIC	0.20	0.14	$$	Electrical signal (binary)	CMOS Electronics
PNP [63]	Mach–Zehnder interferometers, silicon photonics, photodiode, phase shifter	NA	NA	$$	Laser optical pulses	CMOS-compatible photonic chip
*D* ^2^ *NN* [48]	3D printed lenses and optical diffraction	NA	NA	$$	Optical image signal	Free space & Bench-top
AONN [64]	Spatial light modulator, Fourier lens, laser-cooled atom	NA	NA	$$	Optical image signal	Free space & Bench-top
Spiking neurosynaptic network [26]	Phase change material, micro-resonator, and wavelength division multiplexing	NA	NA	$	Laser optical pulses	CMOS-compatible photonic chip
Photonic tensor core [42]	Phase change material, soliton microcombs, SiN micro-resonator, and wavelength division multiplexing	0.4	1.2	$	Soliton frequency comb	CMOS-compatible photonic chip
Optical convolutional accelerator [41]	Soliton microcombs, micro-resonator, Mach–Zehnder modulator, EDFA, and time-wavelength interleaving	1.27	8.061	$$	Electrical waveform	CMOS-compatible photonic chip
PDNN [65]	PIN attenuator, SiGe photodiodes, grating coupler, and microring modulator	2.9	3.5	$$	Optical image signal	CMOS-compatible SOI Photonic chip
PAIM [55]	Meta-surface, optical diffraction, and FPGA	NA	NA	$$$	Optical image signal	Free space & Bench-top
VCSEL-ONN [40]	VCSEL, diffractive optical element, and optical fanout	142.9	6	$$	Amplitude or phase of VCSEL	CMOS-compatible photonic chip
ACCEL [66]	Diffractive optical analog computing, electronic analog computing, SRAM	7.48 + E4	728	$$	Optical image signal	SiO_2_ & CMOS chip (with free space setup)

NA, not available.

## 3 Leveraging 2D materials for advanced photonic neural networks & integrated photonic chips

**Figure 7:**
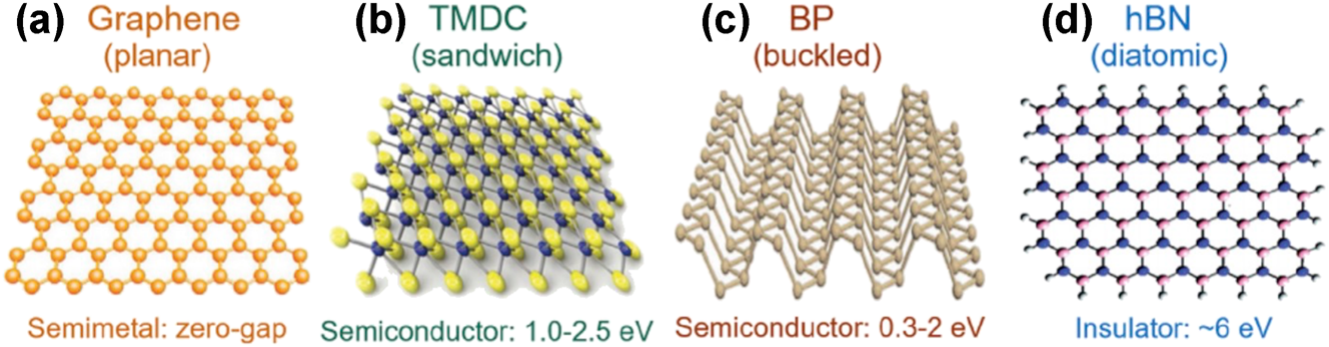
Crystal structures of classic 2D materials: (a) graphene, (b) TMDC, (c) black phosphorus, (d) hexagonal boron nitride [67]. Reprinted from Ref. [67], with permission (CC BY-NC-ND 4.0).

**Figure 8:**
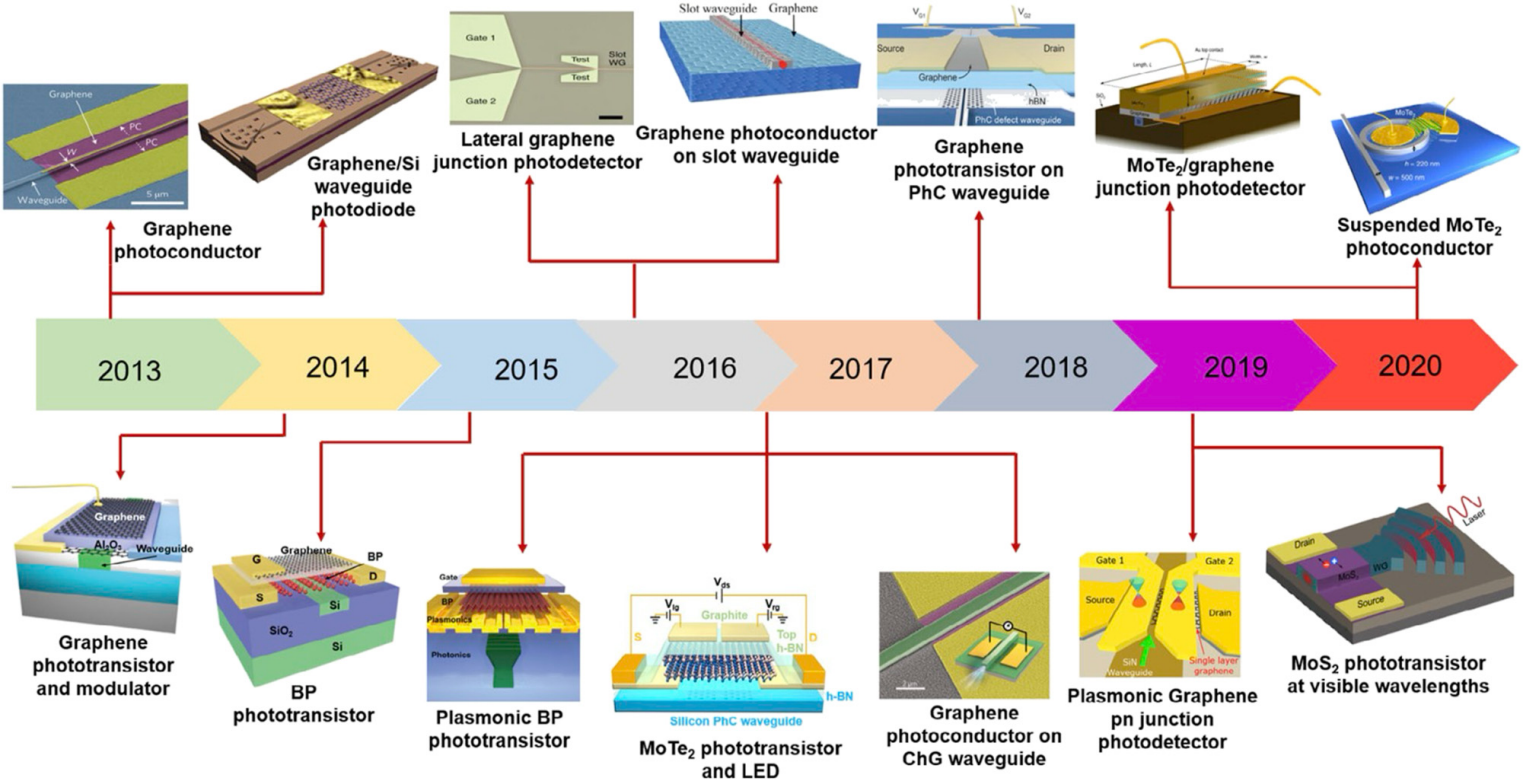
Roadmap of waveguide-integrated photodetectors that are dependent on 2D materials. [68]. Reprinted from Ref. [68], with permission (CC BY 4.0).

As emphasized previously, the rapid evolution of photonic neural networks (PNNs) being applied to AI accelerators is driven by the need for ultrafast, energy-efficient computation. While traditional photonic platforms have demonstrated impressive performance, their scalability and functional diversity are often limited by the intrinsic properties of conventional Si or III-V materials. Given this, two-dimensional (2D) materials such as transition metal dichalcogenides (TMDCs) and graphene (Figure 7) have recently emerged as transformative building blocks for PNNs, providing unique optoelectronic characteristics to overcome the limitations of conventional electronics by harnessing the speed and bandwidth of light for AI computation. 2D materials have been utilized to realize advanced photonic devices such as waveguides and photodetectors (Figure 8). This section focuses on how such 2D materials are being harnessed within PNN architectures, highlighting recent advances that move beyond generic material properties to their concrete roles in photonic AI hardware.

### 3.1 2D Materials as building blocks for PNN components

The exceptional characteristics of 2D materials allow for the creation of key photonic components that enhance the performance and reconfigurability of PNNs in ways such as:

#### 3.1.1 Optical Synapses and Neuromorphic Computing

Graphene and TMDCs are the main 2D materials emerging as critical elements for developing optical synapses, which make up the fundamental building blocks of neuromorphic computing. Unlike conventional electronic synapses, optical synapses make use of light to process information, offering significantly higher speeds and reduced energy consumption.

**a. Graphene-based synapses** Graphene is notable for its ultrafast carrier dynamics and strong light–matter interaction, making it an excellent candidate for optical modulators in emulating synaptic weight updates. Researchers have demonstrated graphene-based synaptic devices capable of mimicking biological synaptic plasticity in both short-term and long-term potentiation. For instance, integrated graphene-based phototransistors are able to directly convert optical stimuli into a ”neural image,” crucial for optical pre-processing in PNNs. Along with this, the ambipolar conductance of graphene allows for both excitatory and inhibitory synaptic behaviors within a single device, and its synaptic plasticity property means it can be dynamically modulated by tuning carrier density [69], [70]. This reconfigurability is crucial for adaptive and subjective perception adjustments in the context of artificial perception systems.

**b. TMDC-based photo-synapses** TMDCs, with their tunable bandgaps and enhanced photoresponsivity, are actively explored for the application of photo-synaptic transistors, especially with their strong light–matter interactions enabling efficient optical modulation of synaptic weights. Hybrid structures with graphene and TMDCs combined can then further enhance broadband detection capabilities and multiwavelength, multilevel optical synaptic memory properties, achieving more than 3 bits of optical memory. This enhanced optical memory facilitates image learning and memory functions for visual simulation in PNNs [69].

#### 3.1.2 All-optical nonlinear activation functions (NAFs)

A significant challenge in PNNs is realizing efficient and compact all-optical nonlinear activation functions, something crucial for complex computations beyond linear operations. 2D materials offer a promising solution due to their strength in nonlinear optical responses.

**Figure 9:**
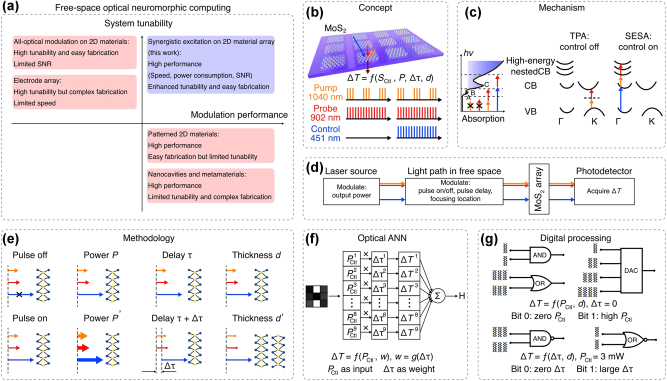
Free-space optical neuromorphic computing concept based on a pure 2D MoS_2_ array. (a) Contradiction between performance and tunability of the system for different strategies. The proposed system shows an improved modulation performance and simultaneously ensures the tunability of the system. (b) Schematic of the computing concept based on a pure MoS_2_ flake array. Within the pumpprobe-control setup (bottom), the relative transmittance (ΔT) is effectively enhanced and modulated, assisting the design of computing functions. (c) Schematic of the computing mechanism. The synergistic transition mainly involves five specific bands: the VB maximum and CB minimum near the K points, the parallel VB and CB between the K and Γ points, and the high-energy nested CBs near the Γ point. The Δ T with the control pulse off is dominated by TPA at the K points. The ΔT with the control pulse on is dominated by SESA between the K and Γ points. (d) Simplified schematic of the computing system. (e) Schematic of the computing methodology. Computing functionalities can be realized by modulating the pulse on/off state, pulse power, pulse delay, and encoding the flake thickness. (f) Schematic of optical ANN. The ΔT is a function of control power and control time delay. The control power in each cell is the input signal, and the control time delay is encoded into weight. The cell thicknesses are identical. (g) Schematic of digital processing functions. Optical AND, OR, NAND, NOR gates, and DAC are realized by encoding the control pulse power and delay into input bits and choosing appropriate cell thickness as input ports [71]. Reprinted from Ref. [71], with permission. Copyright 2024 Nature.

The excellent nonlinear effects and broadband response of 2D materials have been utilized in creating all-optical NAFs directly on-chip. For example, systems utilizing bare molybdenum disulfide (MoS_2_) arrays have properly demonstrated programmable nonlinear optical neuromorphic computing with fast speeds, low energy consumption, and high signal-to-noise ratios (Figure 9). These systems can perform input/weight encoding, vector-matrix multiplication, and output detection, showing the feasibility of free-space optical computing for analog signal processing in PNNs [68], [71]. The tunability of such systems is further enhanced through synergistic encoding of 2D cells and excitation pulses, providing flexibility that is not bound by fixed photonic structures.

#### 3.1.3 In-sensor computing and direct neural processing

**Figure 10:**
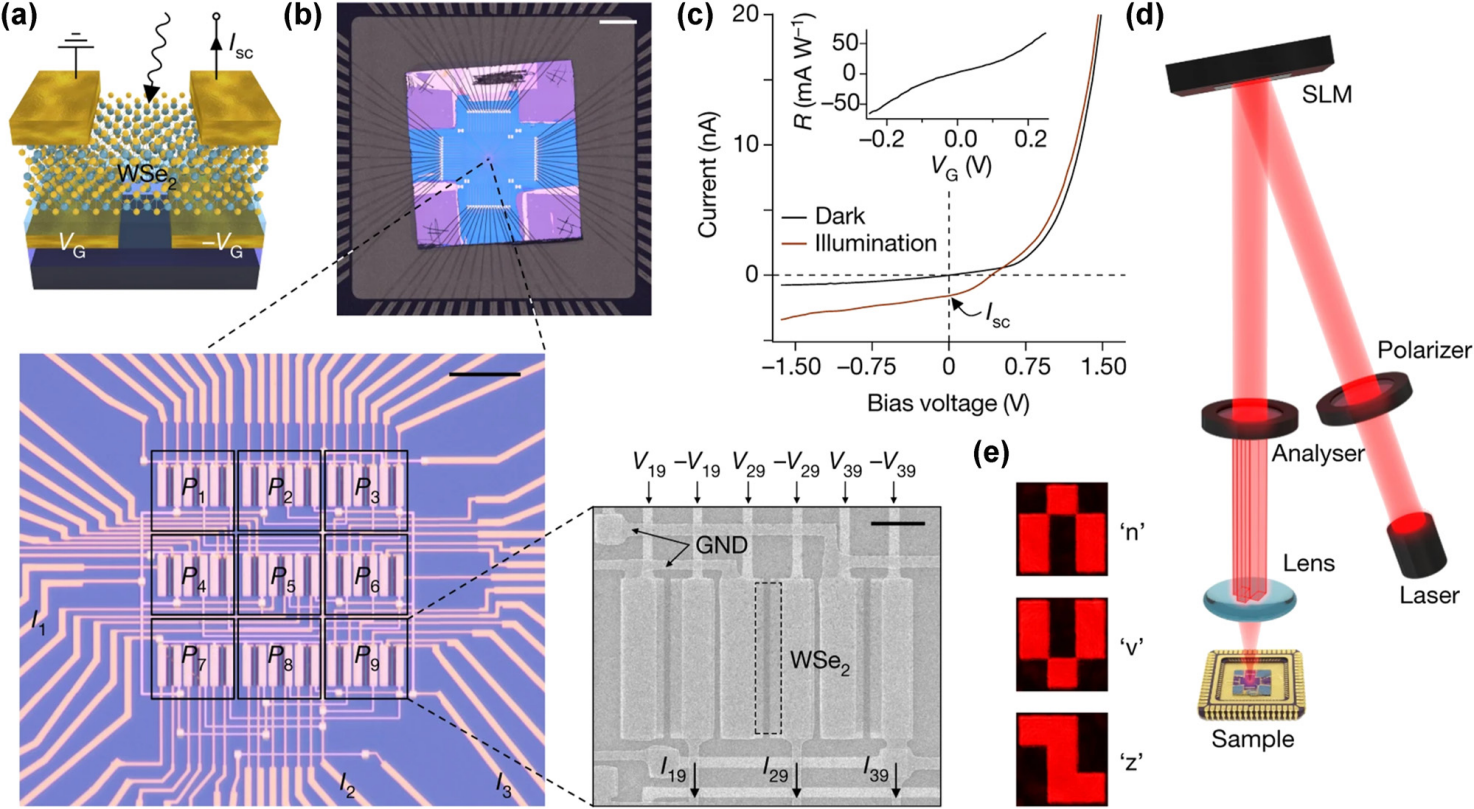
Implementation of the ANN photodiode array built with WSe_2_ photodiodes. The device is operated under short-circuit conditions and the photoresponsivity is set by supplying a voltage pair VG /-VG to the bottom-gate electrodes. (b) Macroscopic image of the bonded chip on the chip carrier. Scale bar, 2 mm. First magnification: microscope image of the photodiode array, which consists of 3 × 3 pixels. Scale bar, 15 µm. Second magnification: scanning electron microscopy image of one of the pixels. Each pixel consists of three WSe_2_ photodiodes/subpixels with responsivities set by the gate voltages. Scale bar, 3 µm. GND, ground electrode. (c) Current–voltage characteristic curve of one of the photodetectors in the dark (blue line) and under optical illumination (red line). The inset shows the gate-voltage tunability of the photoresponsivity. (d) Schematic illustration of the optical setup. Laser light is linearly polarized by a wire-grid polarizer and reflected by a spatial light modulator (SLM). The reflected light is then filtered by an analyser (intensity modulation) and the resulting image is projected onto the photodiode array. (e) Microscope images of the 3 × 3 pixel letters used for training/operation of the network. [72]. Reprinted from Ref. [72], with permission. Copyright 2024 Nature.

Another key innovation enabled by 2D materials in the application of PNNs is the integration of sensing and computation at the hardware level. For example, image sensors based on 2D semiconductors such as WSe_2_ and MoS_2_ can perform both optical image acquisition and neural network inference within the same device, eliminating the latency and energy overhead that results with data transfer between separate sensor/processor units. In a landmark demonstration, a WSe_2_-based photodiode array was configured to act as a neural network, performing supervised and unsupervised learning tasks on optical images directly on the chip (Figure 10). This architecture achieved ultrafast image classification at rates exceeding 20 million bins per second, with the sensor itself constituting the artificial neural network (ANN) and performing both sensing and processing in a single step [72], [73].

In [70], the researchers employed a plasmon-enhanced 2D material neural network to excite localized surface plasmon resonance (LSPR), enhancing the optical signal for improved photodetection. They developed a fully integrated *artificial visual perception and recognition module* (AVPRM) based on a *plasmonic phototransistor array* (PPTA) composed of hybrid two-dimensional materials (MoS_2_/h-BN/WSe_2_). This system achieved high-speed letter classification (500 ns per inference), ultra-low energy consumption per spike (∼2.4 × 10^−17^ J), and an exceptionally wide dynamic range (180 dB). The artificial neural network (ANN) architecture was implemented by encoding pre-trained weights as the drain–source voltage (*V*_DS_) applied to each subpixel.

#### 3.1.4 High-speed optical interconnects and modulators

Efficient data transfer is paramount for PNNs – especially in scaling up large-scale models - and 2D materials excel particularly in creating high-speed, energy-efficient optical interconnects.

**a. Graphene–silicon hybrid modulators** Graphene’s exceptional carrier mobility and broadband absorption as detailed above make it ideal for high-speed optical modulators. Integrating graphene with silicon waveguides has resulted in modulators capable of operating at frequencies exceeding 100 GHz [74]. Such modulators are essential for high-speed data transfer within PNNs and between photonic chips, addressing critical bottlenecks in AI systems. The compact size and low power consumption of these devices further contribute to the energy efficiency required for large-scale AI hardware.

**b. Enhanced interconnects for AI workloads** Companies like black semiconductor are actively developing graphene-based photonic connectivity solutions to enable faster chip-to-chip interconnects. This technology is poised to accelerate training processes for large language models and other AI applications by providing ultra-fast communication pathways within data centers and high-performance computing clusters [75].

### 3.2 Integration strategies for PNNs

**Figure 11:**
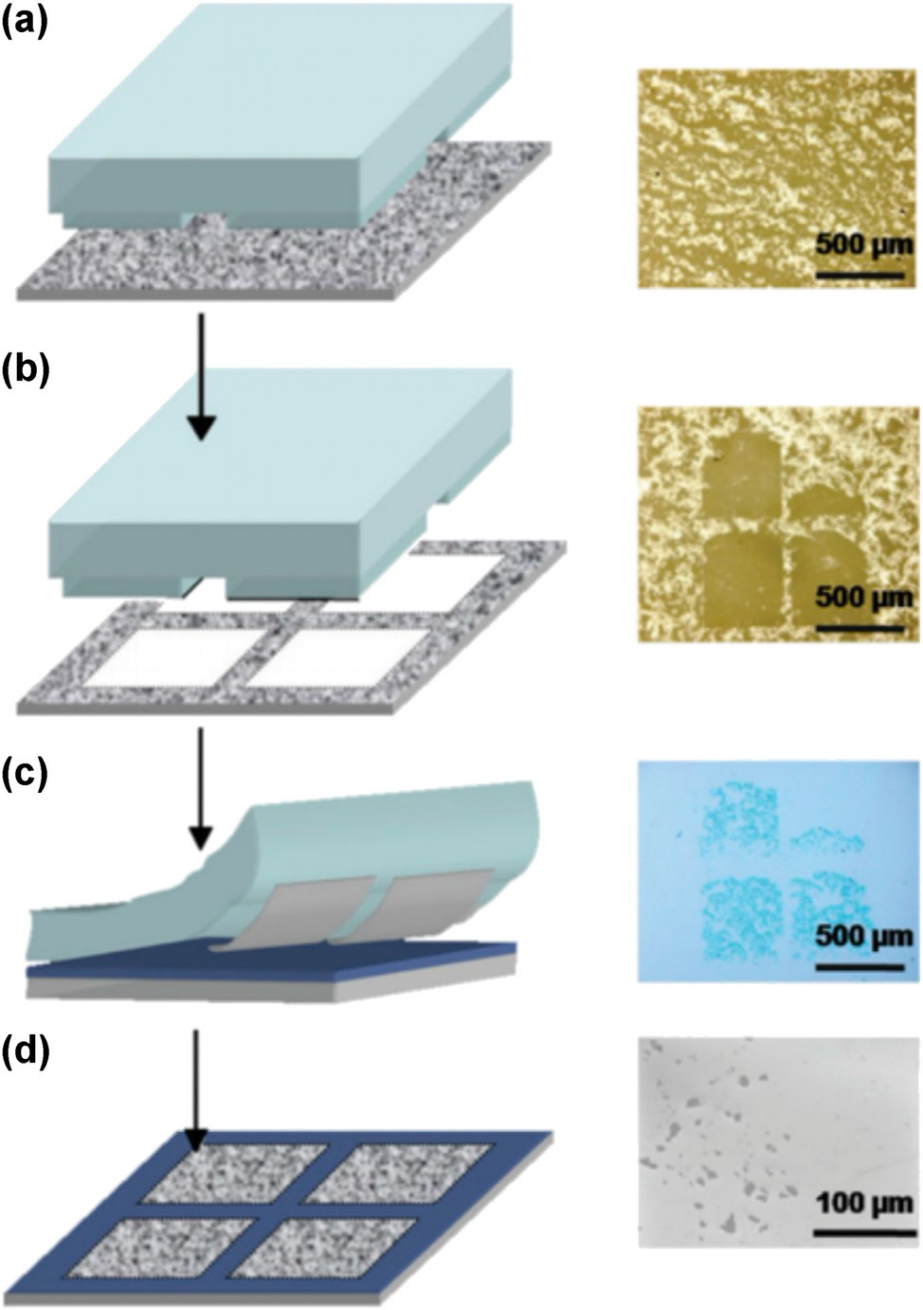
Transfer printing method for 2D materials. Diagram (left) and images acquired from optical microscope (right) showcasing the soft exfoliation and transfer method, one of the main mechanical methods of today. The process follows (a) depositing materials on glass substrate, (b) ink the pre-patterned polydimethylsiloxane (PDMS) stamp carefully, (c) contact inked stamp to heated Si/SiO_2_ substrate, (d) peel away revealing deposited materials [76]. Reprinted from Ref. [76], with permission (CC BY 4.0).

The integration of 2D materials into photonic neural networks often involves sophisticated techniques to preserve their intrinsic properties and enable robust device fabrication:

**Transfer printing** Thin layers of 2D materials are exfoliated and transferred onto silicon substrates without adhesives (Figure 11), preserving intrinsic optical properties of the materials while allowing for precise placement onto photonic structures (waveguides, resonators, etc.) [69].

**Hybrid integration** Combining graphene or TMDCs with existing silicon photonics platforms is another technique, one which enhances light–matter interaction. For example, graphene has been used to create high-speed modulators integrated into microring resonators. These hybrid devices achieve terahertz modulation speeds while maintaining low power consumption [74].

**Figure 12:**
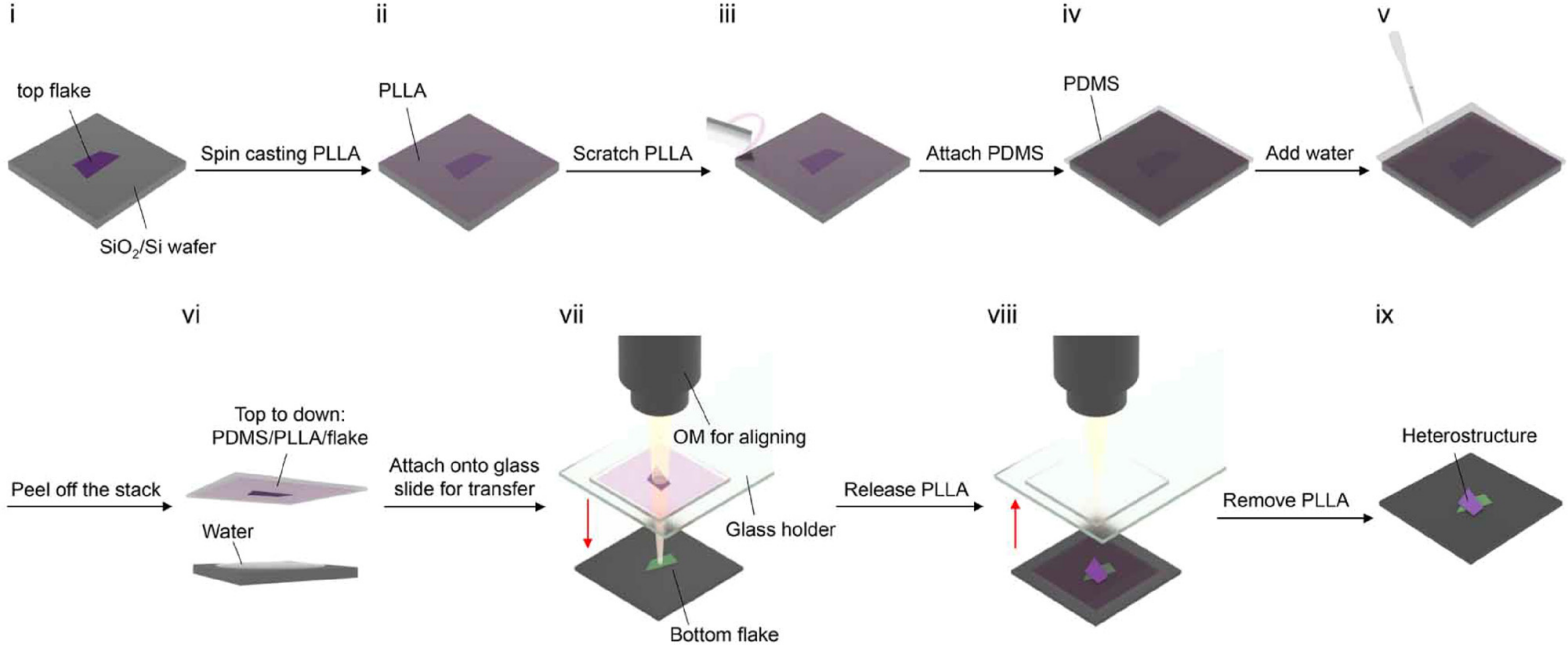
Depiction of a schematic flow of the water immersion method used for constructing Van der Waals heterostructure without etchant [76]. Reprinted from Ref. [76], with permission (CC BY 4.0)

**Van der Waals heterostructures** For this technique, stacking different 2D materials enables the creation of heterostructures with tailored optical properties (Figure 12), such as tunable bandgaps and anisotropic refractive indices. These heterostructures are viewed to be useful for waveguiding applications where confinement factors need optimization [67], [76], [77].

### 3.3 Outlook and challenges

The integration of 2D materials holds immense promise for the advancement of PNNs, enabling new levels of speed, energy efficiency, and reconfigurability. However, several challenges must be addressed for widespread adoption:

**Scalability and manufacturing** The delicate nature of ultrathin 2D materials poses challenges during large-scale manufacturing, and advances in transfer printing techniques and wafer-scale synthesis are needed to overcome this limitation to properly make this technology scalable [76].

**Material stability** Some 2D materials, including both graphene and TMDCs, degrade under ambient conditions, and for this technology to catch on, there needs to be development of protective coatings, encapsulation techniques, or general preservation advancements for long-term reliability [78].

**Integration complexity** Achieving seamless integration with existing CMOS processes requires further optimization for varying techniques and interface engineering before this new technology can be properly integrated into the general world [77].

Future research should focus on addressing these challenges while continuing to explore new material systems that complement not only graphene and TMDCs but also black phosphorus. With both of these paths combined, the development of hybrid platforms combining electronic, photonic, and 2D material-based components seem promising in paving the way for transformative advancements in AI hardware and technology for the near future.

### 3.4 Comparison with existing PNNs

In this section, we incorporate more specific quantitative key performance indicators (e.g., *TOPS*/*J*, *TOP*/*mm* ^2^/*s* etc.) from cited literature for specific 2D material-enabled photonic computing architectures and show direct comparisons to existing PNN architectures (e.g., VCSE-ONN and ACCEL). See this detailed comparison in Table 2. We can see that 2D materials already provide energy efficiencies and latencies similar to existing PNNs. Some compute densities were not calculated because the chip size wasn’t reported in the literature.

**Table 2:** Comparison of state-of-the-art PNNs to next-gen Photonic Solutions enabled by 2D materials (bold-font), in terms of energy efficiency, compute density, signal transmission media, and latency.

Name abbrv.	Energy efficiency (*TOP*/*J*)	Compute density (*TOP*/*mm* ^2^/*s*)	Signal transmission medium	Latency
Photonic tensor core [42]	0.4	1.2	Soliton frequency comb	NA
VCSEL-ONN [40]	142.9	6	Amplitude or phase of VCSEL	< 1fs per symbol in principle, negligible compared to the integration [40]
ACCEL [66]	7.48 + E4	728	Optical image signal	72 ns for each frame
**PPTA** [70]	4.17 + E4	NA	Electrical signal (photocurrent)	500 ns
**2D** **MoS_2_** **array** [71]	0.1–0.2	NA	Laser optical pulses	5.47 ps
**ANN photodiode array** [72]	self-powered (photovoltaic device)	0.07	Optical image signal	50ns

## 4 Spintronics for photonic neuromorphic computing chips

Current AI computing solutions using CMOS circuits or even emerging memristors either lack essential neuromorphic characteristics or suffer from limited endurance and stochastic control. This hardware-algorithm gap fundamentally restricts neuromorphic computing’s potential to achieve brain-like efficiency and adaptability.

Nano-photonics, as an emerging interdisciplinary subject, integrates the principles of nanotechnology and photonics, aiming to explore and harness the manipulation of light wave by nanoscale structures. In the landscape of photonics, active devices and passive devices are crucial and have broad application prospects. Spintronics, also known as spin electronics, is a field of study that explores the use of an electron’s intrinsic spin and magnetic moment, in addition to its charge, in solid-state devices. It focuses on manipulating and controlling electron spins for data storage, processing, and other functionalities, potentially leading to more efficient and powerful electronic devices.

Neuromorphic systems aim to mimic the computational and cognitive capabilities of the brain by leveraging the principles of neural networks. This section systematically investigates the synergistic integration of spintronic devices with nanophotonic architectures for neuromorphic computing. We first introduce the basic spintronic devices and explain their basic principles and how to achieve their functions. Then we explored the possibilities and prospects of opto-spintronic devices in the context of neuromorphic computing under the background of optoelectronic fusion.

### 4.1 Key spintronic technologies for neuromorphic computing

Spintronic devices exhibit unique advantages that position them as leading candidates for neuromorphic computing hardware. Their intrinsic nonvolatility, ultrafast dynamics, and near-unlimited endurance enable energy-efficient and biologically plausible neural network implementations [13]. Crucially, spintronic technologies leverage magnetic and spin-based phenomena to natively emulate neuro-synaptic functionalities while maintaining compatibility with conventional CMOS manufacturing processes. Three core advantages drive their prominence: (1) Stochasticity in magnetization switching and spin precession mirrors probabilistic neural firing mechanisms, enabling event-driven spiking neural networks (SNNs) with sparse coding efficiency [79]; (2) Multistate magnetization dynamics (e.g., domain wall motion, skyrmion nucleation); provide analog memristive behavior essential for synaptic weight modulation [80]; and (3) Nonvolatile state retention eliminates static power consumption during idle periods [11]. These attributes address critical von Neumann bottleneck limitations while surpassing competing memristive technologies in speed and reliability. 

**Figure 13:**
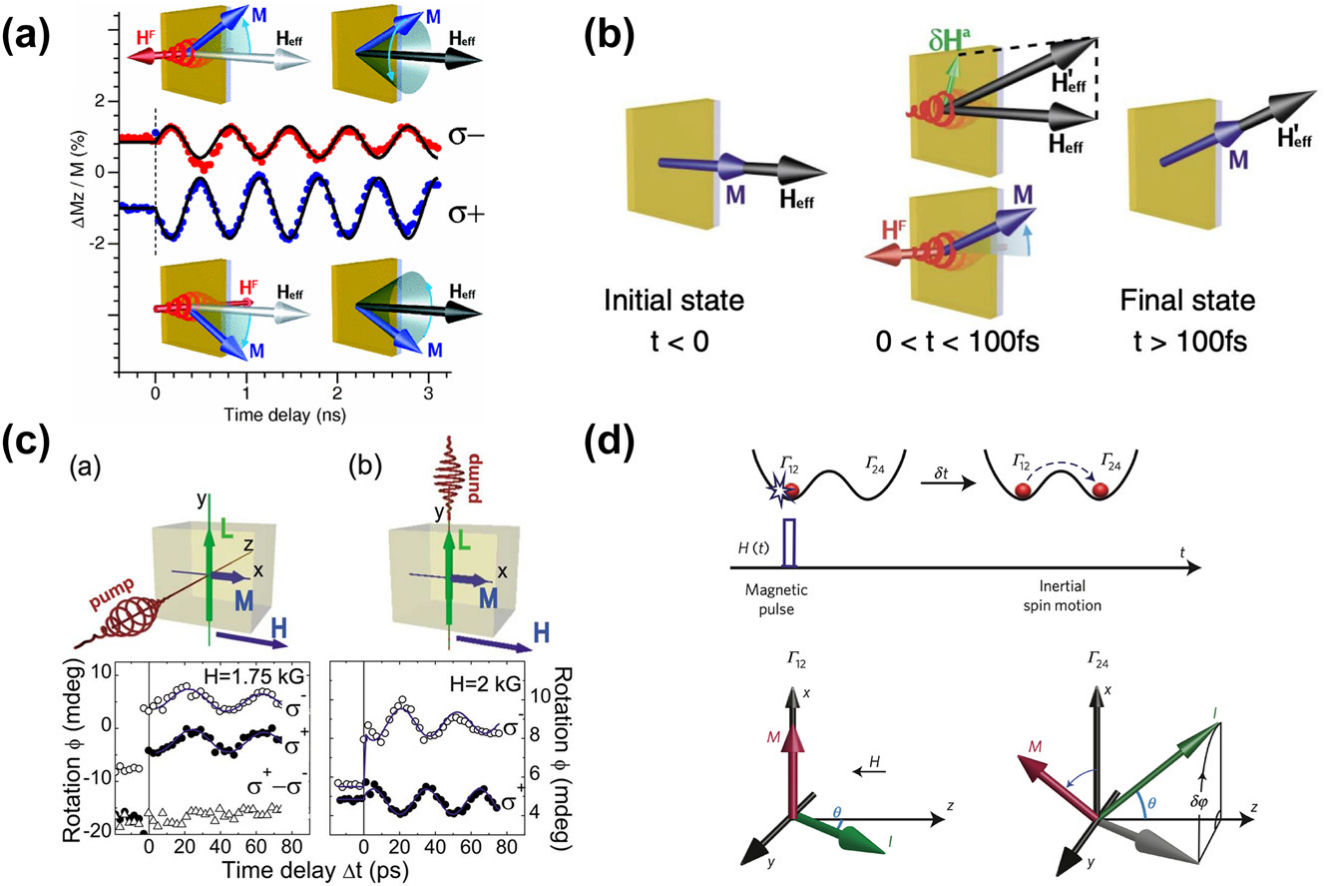
Optically controlled spintronic devices: (a) Circularly polarized light drives magnetic moment precession (garnet film). Precession of the magnetization following excitation with circularly polarized light. The two helicities *σ* ^+^ and *σ* ^−^ give rise to precession with opposite phase and different amplitude [87]. (b) Graphical illustration of the switching process: Initially at *t* < 0 the magnetization is along **H**_eff_. During the presence of the laser pulse 0 < *t* < 100 fs photoinduced modification of the anisotropy fields leads to a new metastable equilibrium along $H_{\text{eff}}^{\prime }$ [87]. (c) Spin precession excited by circularly polarized pump pulses propagating along the *z* axis and along the *y* axis. *σ* ^+^ − *σ* ^−^ is the difference between the spin precession amplitude excited by right- and left-handed circularly polarized pump pulses [88]. (d) Spin switching in HoFeO_3_, Γ_12_ and Γ_24_ are the two metastable phases present between 38 and 52 K. *H* represents an effective magnetic-field pulse that initiates an inertial motion of spins from the Γ_12_ to the Γ_24_ magnetic phase [89]. (a-b) are reprinted from Ref. [87], with permission. Copyright, 2024 American Physical Society; (c) is reprinted from Ref. [88], with permission. Copyright, 2024 American Physical Society; (d) is reprinted from Ref. [89], with permission. Copyright, 2024 Nature.

The magnetic tunnel junction (MTJ) constitutes the foundational spintronic building block, demonstrating versatile neuromorphic functionality through two operational regimes. In superparamagnetic mode, stochastic switching between parallel and antiparallel states generates Poisson-distributed spikes for probabilistic computing, achieving 604 % tunneling magnetoresistance (TMR) ratios in CoFeB/MgO structures [81]. Magnetic tunnel junctions are currently being developed for nonvolatile memory in the back end of complementary metal oxide semiconductor (CMOS) chips, and commercial foundries have already incorporated these devices into their processes. This compatibility, coupled with the ability to achieve multiple functions by changing the geometry, makes magnetic tunnel junctions an attractive candidate for the development of efficient computing. When configured as spin-torque nano-oscillators (STNOs), MTJs produce GHz-range voltage oscillations that synchronize with external stimuli, enabling coupled oscillator networks for pattern recognition [82]. Spin–orbit torque (SOT) devices extend these capabilities through field-free magnetization switching in heavy metal/ferromagnet bilayers. SOT-driven spin Hall nano-oscillators (SHNOs) achieve mutual synchronization in 2D arrays [83], while three-terminal MTJs separate read/write paths for enhanced synaptic precision [84]. Domain wall motion in magnetic nanowires provides continuous resistance modulation ideal for analog synapses [80].

Emerging topological spin textures like magnetic skyrmions offer particle-like dynamics for bio-inspired computing paradigms. Skyrmion nucleation and annihilation in chiral magnets (¡ 10 µms diameter) emulate neurotransmitter release probabilities [85]. Antiferromagnetic (AFM) spintronics introduces terahertz-range dynamics and stray-field immunity, enabling dense crossbar arrays through compensated magnetic moments [86]. Integration of these technologies enables all-spin neural networks combining STNO-based neurons [82], domain wall memristive synapses [11], and skyrmionic probabilistic interconnects [85] – a hardware ecosystem addressing the memory-processor dichotomy through physics-level co-design.

### 4.2 Optically-controlled spintronic in-Memory computing units and photon-spin interconnect networks

In this section, we discuss some recent advances in optically controlled spintronic devices and how they have enabled novel non-von Neumann architectures for neuromorphic computing. We provide some additional context in Section 7.5. Breakthrough demonstrations by Hansteen, Fredrik et al. [87] and Kalashnikova et al. [88] revealed two complementary pathways for ultrafast optical control of magnetic order. Circularly polarized femtosecond pulses generate 20-T effective magnetic fields via the *inverse Faraday effect*, transferring photon angular momentum to spins through spin–orbit coupling within 100 fs (Figure 13a and b). This enables room-temperature excitation of 0.6-T photomagnetic fields in Gd_3_Fe_5_O_12_ garnet films and drives 200-GHz spin precession in DyFeO_3_ antiferromagnets [90]. Concurrently, linearly polarized pulses reconfigure magnetic anisotropy through the *inverse Cotton-Mouton effect*, where the electric field vector direction controls magnetic easy-axis orientation. This non-thermal mechanism achieves deterministic in-plane switching of magnetic precession planes in FeBO_3_ crystals in under 1 ps (Figure 13c). These dual approaches collectively establish optical polarization as a universal control parameter for magnetic systems, eliminating external field requirements (Table 3) while achieving switching speeds three orders of magnitude faster than conventional methods. A concurrent breakthrough in HoFeO_3_ antiferromagnets demonstrates *inertial magnetic switching* driven by femtosecond pulses (Figure 13d) [89]. This mechanism exploits light-triggered angular momentum transfer to accelerate spins beyond Landau–Lifshitz–Gilbert equation constraints, achieving sub-picosecond magnetization reversal without pre-magnetization requirements. Critically, the threshold-driven switching dynamics (10 fJ/operation) directly emulate biological neuronal firing mechanisms, implementing hardware-level ”integrate-and-fire” functionality while consuming three orders of magnitude less energy than CMOS synapses (Table 3). Building on these foundations, magnon coherent control enables quantum-level manipulation of spin waves for neuromorphic information processing. Phase-locked femtosecond pulse pairs demonstrate constructive/destructive interference of magnons in TmFeO_3_, implementing spike-timing-dependent plasticity (STDP) learning rules with 0.1-rad phase precision [91]. This provides physical hardware realization of temporal coding in spiking neural networks, where magnon phase coherence directly encodes synaptic weight updates. Multistate optically controlled magnetic anisotropy in ferromagnetic semiconductors (e.g., Ga_1-x_Mn_*x*_As) creates reconfigurable synaptic weights through polarization-dependent resistance states. Linearly polarized pulses modify magnetocrystalline anisotropy to establish > 4 distinct resistance levels, enabling analog weight storage with ns-scale reconfiguration [92]. This mimics biological synaptic plasticity while achieving 10 × higher energy efficiency than CMOS-based neuromorphic systems. Nonvolatile optically controlled magnetic phase transitions in alloys like FeRh provide memory-processor integration through femtosecond-induced AFM → FM transitions [93]. The sub-ps switching (<300 fs) and inherent nonvolatility emulate neuronal ”integrate-and-fire” dynamics while maintaining state retention, crucial for implementing reservoir computing architectures. Energy consumption of < 10 fJ/bit surpasses biological synapses by two orders of magnitude. These advances collectively establish photon-spin networks as platforms for event-driven neuromorphic computation, combining < 100-fJ/op energy efficiency with THz-bandwidth communication through optically excited spin-wave interconnects. Critical challenges remain in scaling interconnect densities below 100 nm via plasmonic focusing while maintaining phase coherence across > 10^3^ node networks. All in all, photonic-spintronic systems have proven to be beneficial for next-gen AI computing hardware for LLMs.

**Table 3:** Comparing next-gen optically-controlled spintronics enabling neuromorphic computing to traditional CMOS and photonic solutions, showcasing main advantages of opto-spintronic systems.

Traditional component being replaced	Next-gen opto-spintronic technology	Benefits and advantages of opto-spintronics
External magnetic fields	Inverse Faraday effect and inverse Cotton–Mouton effect	Using circularly or linearly polarized femtosecond laser pulses to generate effective fields or alter magnetic anisotropy, completely eliminating the need for external electromagnets or current-generated fields. Simplifies device architecture and enables ultrafast control (<1 ps) [88].
CMOS transistors/lasers/PCMs for neurons	Inertial magnetic switching	In antiferromagnets (e.g., HoFeO_3_), femtosecond pulses trigger deterministic magnetization reversal via angular momentum transfer. Threshold-driven dynamics emulate biological neuronal ”integrate-and-fire” mechanism at hardware level with low energy (10 fJ/operation) [89].
CMOS/Memristors for synaptic weights	Multistate optical magnetic anisotropy	Linearly polarized light modifies magnetocrystalline anisotropy in ferromagnetic semiconductors (e.g., Ga_1-x_Mn_*x*_As), establishing > 4 distinct nonvolatile resistance states [92]. Enables analog synaptic weight storage with high energy efficiency.
Separate memory and processing units	Nonvolatile optical magnetic phase transitions	Femtosecond laser pulses induce AFM to FM phase transition in alloys (e.g., FeRh). Switching is ultrafast (<300 fs) and nonvolatile [93]. Enables in-memory computing, avoiding data movement overhead.
Electronic/photonic interconnects	Optically excited spin-wave interconnects	Spin-wave transmission offers THz-bandwidth communication with low energy consumption (<100 fJ/operation), replacing or complementing traditional copper interconnects [93].

## 5 Principles of transformer neural networks and LLMs and their relations to photonic computing

### 5.1 Transformer architecture

**Figure 14:**
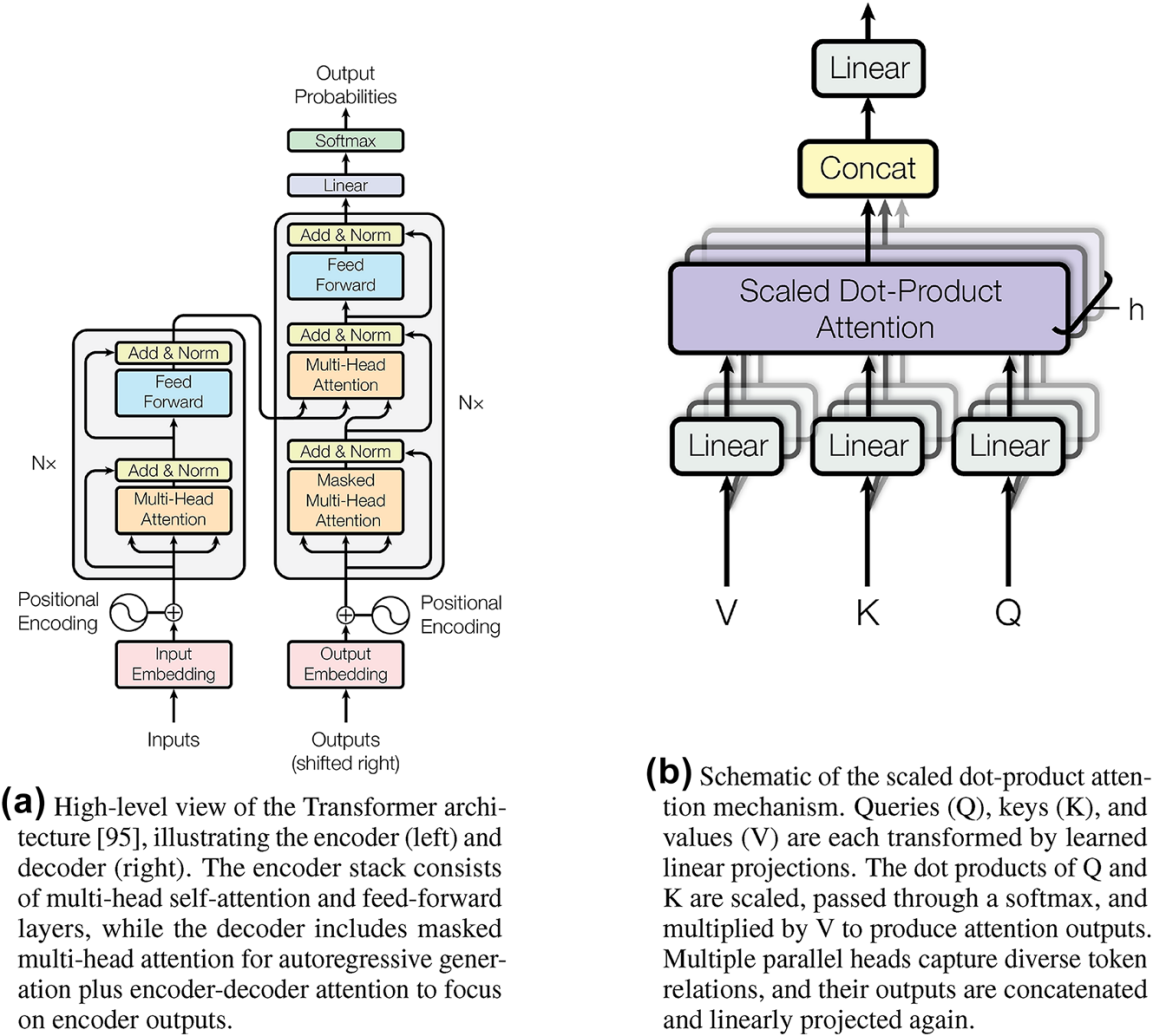
Transformer neural networks used in modern LLMs. Reproduced with permission [94], CC BY 4.0. (a) High-level view of the Transformer architecture [94], illustrating the encoder (left) and decoder (right). The encoder stack consists of multi-head self-attention and feed-forward layers, while the decoder includes masked multi-head attention for autoregressive generation plus encoder-decoder attention to focus on encoder outputs. (b) Schematic of the scaled dot-product attention mechanism. Queries (Q), keys (K), and values (V) are each transformed by learned linear projections. The dot products of Q and K are scaled, passed through a softmax, and multiplied by V to produce attention outputs. Multiple parallel heads capture diverse token relations, and their outputs are concatenated and linearly projected again.

Existing LLMs are all based on a DNN proposed by Vaswani et al. [94] who introduced sequence modeling by relying on an attention mechanism instead of recurrence or convolution, which is now widely known as the *Transformer* architecture [94] (Figure 14). In the original Transformer design for machine translation [94], an encoder–decoder structure was employed. The encoder stack processes the input through self-attention layers – which allow each token to attend to others in the sequence – followed by position-wise feed-forward networks. The decoder stack then generates output tokens using a self-attention mechanism combined with encoder–decoder attention to focus on the encoder’s output [94]. A high-level illustration of this architecture, along with the underlying scaled dot-product attention mechanism, is shown in Figure 14. This design enables the model to handle sequences without maintaining an RNN-style hidden state, thereby improving parallelization during both training and inference [94]. With this architecture, the Transformer achieved superior translation quality while requiring significantly less time to train compared to prior recurrent or convolutional models [94].

The key innovation behind the transformer is *self-attention*. This mechanism helps build contextualized representations by allowing each position in the sequence to selectively attend to other positions. Another essential idea is *multi-head attention*, which is used to capture different aspects of token relations [94]. Each attention head learns to focus on different patterns, enabling the model to integrate diverse information about word relationships by combining the outputs from multiple heads [94]. This attention mechanism gives the transformer the ability to process long sequences effectively; since any token can influence any other through weighted attention, it addresses long-range dependencies more robustly than the fixed-step interactions of RNNs. Furthermore, transformers are highly scalable to long sequences because attention across all positions can be computed in parallel. In contrast, recurrent neural networks must process tokens sequentially.

Another important concept in transformers is the incorporation of *positional encodings* to inject information about token positions into the model. The original approach used fixed sinusoidal position embeddings added to token embeddings [94]. These positional signals help the model understand the ordering of words (e.g., distinguishing “Alice answered Bob” from “Bob answered Alice”). After embedding the inputs and adding positional encodings, each transformer layer applies layer normalization and residual skip connections around its sub-layers. The residual connections mitigate vanishing gradient issues by adding the layer’s input to its output, while layer normalization ensures that activations remain well-conditioned.

Together, the Transformer architecture – comprising multi-head self-attention, feed-forward networks, residual/normalization layers, and positional encodings – provides a highly parallelizable and effective approach to modeling sequences. It quickly became the dominant architecture in natural language processing, enabling the training of much larger models than was feasible with RNNs or CNNs, thanks to its ability to capture long-range context and process entire sequences in parallel [95].

### 5.2 Memory integration for long-term context

In the early stage, transformer models were limited to input sequences of only a few thousand tokens due to the constraints of positional encoding schemes and the *O*(*n* ^2^) memory cost of self-attention. However, long-context reasoning in advanced large language models requires architectural innovations to handle long input sequences efficiently. Such innovations include enhanced positional encodings (e.g., ALiBi and Rotary embeddings) as well as efficient attention algorithms like FlashAttention.

**Positional Encoding and Extrapolation:** Transformers require a mechanism to encode the position of each token since the model itself is order-agnostic. Standard methods – such as fixed sinusoidal embeddings – have a fixed context limit and often struggle to generalize beyond the training length. *ALiBi* (Attention with Linear Biases) is a technique that enables extrapolation to longer sequences without retraining by dispensing with absolute positional embeddings. Instead, ALiBi adds a fixed penalty proportional to the distance between the query and key tokens, introducing a linear bias in the attention scores [96]. The attention mechanism naturally down-weights far-distance tokens but never entirely ignores them. Press et al. [96] showed that a model trained with ALiBi on 1K-token sequences can generalize to 2K or more tokens during inference, achieving performance comparable to a model trained on longer sequences. In essence, ALiBi allows long-context reasoning without a fixed positional index limit, as tokens beyond the training length simply receive larger bias values rather than entirely novel embedding vectors.

Another popular approach is *rotary position embedding (RoPE)* [97]. RoPE encodes positional information by rotating the query and key vectors within each attention head using a rotation matrix defined by sinusoidal frequencies. This rotation angle increases with the token’s position, enabling the inner product of rotated queries and keys to depend solely on their positional difference rather than on absolute positions. Consequently, the model is able to process sequences longer than those encountered during training. Empirically, models using RoPE have been scaled from 2K to 8K or even 16K-token contexts through interpolation methods. Jianlin et al. [97] demonstrated that models incorporating RoPE achieved improved performance on long-sequence benchmarks compared to alternative approaches.

**Efficient Attention Computation:** Even with enhanced positional encoding, the standard self-attention mechanism has a quadratic *O*(*n* ^2^) memory cost when processing large amounts of tokens. *FlashAttention* [98] is an exact attention computation method optimized to minimize memory reads and writes, effectively making the computation I/O-bound rather than memory-bound. FlashAttention introduces a tiling strategy to store intermediate results in high-speed on-chip memory, dramatically reducing the need for expensive GPU memory access. Thus, while naive self-attention scales quadratically with sequence length, FlashAttention uses memory linear in the sequence length and achieves significant speedups.

Another recent technique is NTK (Neural Tangent Kernel)-aware interpolation, which “stretches” the rotary positional embeddings during inference. This enables an extension of the context length without retraining. Alibaba’s Qwen-7B/14B models, for example, utilize NTK-aware interpolation, log-scaled attention, and local window attention to achieve context lengths well beyond 8K tokens [99]. The underlying principle of RoPE is that when frequencies are not modified, extending the trained context beyond boundaries results in angles that the model has never encountered. However, NTK interpolation ensures the rotation angles are adjusted within a range that the model is familiar with the new maximum length. This stretching technique has been empirically demonstrated in code-generation models (e.g., CodeLlama was extended from 16K to 100K with minimal performance degradation using a similar approach).

Advanced LLMs employ several mechanisms to enable long-context reasoning. These include positional encoding techniques that facilitate extrapolation – such as ALiBi’s distance-based linear bias and RoPE’s rotational encoding – and efficient attention algorithms like FlashAttention that overcome the quadratic memory bottleneck. In combination, these mechanisms have significantly increased the effective context length from roughly 1K to as high as 100K tokens, thereby expanding the range of applications from reading long contracts and logs to maintaining coherent context over vast document collections. Long-context architectures are thus crucial for bringing LLMs closer to human-like long-term coherence in conversation and writing.

### 5.3 Computational operations in transformer-based LLMs

A modern large language model employs a fundamental set of tensor operations that are iteratively applied across layers. These operations encompass linear projections, scaled dot-product attention calculations (typically involving multiple heads in parallel), normalization layers, and position-wise feed-forward transformations. We provide a comprehensive exposition of these operations, with a particular focus on the mathematical formulation of the attention mechanism and the associated computations.

**Scaled Dot-Product Attention** The core of transformers lies in its scaled dot-product attention mechanism. Given a set of *n* input tokens, we first project each token’s embedding (of dimension *d*_model) into three learned representations: queries *Q*, keys *K*, and values *V*. Given a set of query vectors *Q*, key vectors *K*, and value vectors *V* (each row corresponding to a sequence element), the *scaled dot-product attention* is defined as in Eq. (1) [94].

(1) $$\text{Attention}\left(Q,K,V\right)=\mathrm{softmax}\left(\frac{QK^{T}}{\sqrt{d_{k}}}\right)V$$

In this formulation, the matrix product *QK* ^*T*^ yields an (*n* × *n*) score matrix for *n* query and *n* key vectors. The scaling factor $1/\sqrt{d_{k}}$ is used to prevent the dot products from growing too large in magnitude when *d*_*k*_ is high. The softmax function is then applied row-wise to normalize each row of the score matrix into a probability distribution, producing the attention weights.

These attention weights are used to compute a weighted sum of the value vectors in *V*, resulting in the final attended output. Intuitively, each output vector is a context-dependent mixture of all input values, where the mixing weights reflect the relevance of each key vector to the current query.

Equation (1) is a core operation in transformer models [94], enabling each position in a sequence to attend to (i.e., selectively focus on) information from other positions based on content similarity.

**Figure:**
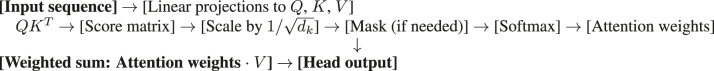


In practice, *Q*, *K*, and *V* are computed by learned linear projections of the input embeddings or hidden states. Let $X\in R^{n\times d_{\text{model}}}$ be the matrix of *n* input vectors (each of dimension *d*_model_). Then:

$$Q=XW^{Q},\;K=XW^{K},\;V=XW^{V}$$

where $W^{Q},W^{K}\in R^{d_{\text{model}}\times d_{k}}$ and $W^{V}\in R^{d_{\text{model}}\times d_{v}}$ are learned projection matrices.

The masking step is applied in settings such as autoregressive self-attention, where a given position must not attend to future tokens. This is typically implemented by adding a large negative bias (e.g., −*∞*) to disallowed entries in *QK* ^*T*^ prior to softmax. As a result, those positions receive zero attention weight.

This creates a triangular masking pattern that enforces causality in sequence generation, ensuring that each token’s representation is influenced only by current and past tokens. After masking (if applied) and softmax, the multiplication with *V* completes the attention computation.

**Multi-head attention** Instead of performing a single attention operation, transformers utilize *multi-head attention* [94], employing *h* parallel attention heads to capture information from multiple representation subspaces simultaneously. Given an input matrix $X\in R^{n\times d_{\text{model}}}$ representing *n* token embeddings, the model computes distinct sets of learned linear projections to generate query, key, and value matrices for each head independently:

(2) $$Q_{i}=XW_{i}^{Q},\;K_{i}=XW_{i}^{K},\;V_{i}=XW_{i}^{V},\;i=1,\ldots ,h$$

Here, $W_{i}^{Q},W_{i}^{K},W_{i}^{V}\in R^{d_{\text{model}}\times d_{k}}$ are learned projection matrices. Each head *i* then computes its output as

$${\text{head}}_{i}=\text{Attention}\left(Q_{i},K_{i},V_{i}\right)$$

where the attention function is as defined in Eq. (1). These *h* output matrices, each of dimension *n* × *d*_*k*_, are subsequently concatenated and projected through another learned linear transformation $W^{O}\in R^{\left(h\cdot d_{k}\right)\times d_{\text{model}}}$ to produce the final output of the multi-head attention mechanism:

(3) $$\text{MultiHead}\left(X\right)=\left[{\text{head}}_{1};{\text{head}}_{2};\ldots ;{\text{head}}_{h}\right]W^{O}$$

where $\left[\cdot \;\cdot \;\cdot \right]$ denotes concatenation along the last dimension.

The use of parallel attention heads allows the model to attend simultaneously to different kinds of relationships and semantic subspaces within the token sequence [94]. Practically, implementing multi-head attention involves specific tensor reshaping and transposition operations. For instance, after computing a combined linear projection *XW* ^*Q*^ with shape (*n*, *h* ⋅ *d*_*k*_), it is reshaped to (*n*, *h*, *d*_*k*_) and transposed to (*h*, *n*, *d*_*k*_) to facilitate parallel and independent computation across heads. Identical transformations are applied to the *K* and *V* projections.

These tensor reshaping steps rearrange the memory layout to optimize data flow, preparing data efficiently for batched matrix multiplications – first computing the *QK* ^*T*^ dot-product scores independently within each head, and subsequently applying attention weights to the corresponding value vectors [100]. Such operations are crucial for efficiently leveraging highly parallel hardware architectures like GPUs and TPUs.

**Layer Normalization** Transformers employ *layer normalization* [101] after the multi-head attention and feed-forward sub-layers (described above and below) to stabilize training and improve convergence. Layer normalization operates by rescaling and recentering the components of each token’s activation vector individually, ensuring zero mean and unit variance, followed by learned linear shifts.

Formally, given an input vector *x* = (*x*_1_,…, *x*_*d*_) – such as the activations for a single token at a specific layer – the layer normalization is defined as:

(4) $$\begin{array}{ll} y_{i} & =\text{LayerNorm}{\left(x\right)}_{i}=\gamma_{i}\cdot \frac{x_{i}-\mu }{\sqrt{\sigma^{2}+\epsilon }}+\beta_{i},\\ & \;\;\;\;\;\;\;\text{for }i=1,\ldots ,d \end{array}$$

where *μ* and *σ* ^2^ represent the mean and variance of the vector components:

$$\mu =\frac{1}{d}\overset{d}{\underset{j=1}{\sum }}x_{j},\;\sigma^{2}=\frac{1}{d}\overset{d}{\underset{j=1}{\sum }}{\left(x_{j}-\mu \right)}^{2}$$

Here, *γ*_*i*_ and *β*_*i*_ are learnable parameters (gain and bias) introduced to enable the model to adaptively scale and shift the normalized activations, respectively. The constant *ϵ* is a small positive value included for numerical stability, preventing division by zero.

Critically, layer normalization is applied independently across the feature dimensions for each token, not across different tokens in the sequence. This design choice allows highly parallel implementation across sequence positions, significantly accelerating computation. The entire operation can be efficiently computed through basic vectorized arithmetic primitives: mean and variance computation, followed by element-wise subtraction, division, scaling, and shifting [94], [101].

**Position-wise feed-forward network** Following the attention sub-layer, each Transformer block includes a fully-connected *position-wise feed-forward network (FFN)* [94]. This network is applied independently to each token and consists of two linear transformations separated by a non-linear activation function. Mathematically, given an input token representation $x\in R^{d_{\text{model}}}$ , the FFN computes:

(5) $$\text{FFN}\left(x\right)=W_{2}\;\sigma \left(W_{1}x+b_{1}\right)+b_{2}$$

where $W_{1}\in R^{d_{\text{ff}}\times d_{\text{model}}}$ and $W_{2}\in R^{d_{\text{model}}\times d_{\text{ff}}}$ are learned weight matrices, and *b*_1_, *b*_2_ are learned bias vectors. The intermediate dimension *d*_ff_ is typically larger than *d*_model_ – for example, *d*_ff_ = 4 *d*_model_ in many standard transformer architectures [94], [102] – to enhance representational power.

A widely-used activation function in transformer FFNs is the *Gaussian error linear unit (GeLU)* [103], defined as:

(6) GeLU(z)=z⋅Φ(z)

where Φ(*z*) is the cumulative distribution function (CDF) of the standard Gaussian distribution. GeLU is a smooth, continuous non-linear activation popularized by the BERT model [102] for its empirical effectiveness. In practice, GeLU is computed efficiently via approximate analytical expressions involving elementary functions such as tanh or the error function erf.

Although alternative activations such as ReLU or SiLU (Swish) may also be employed, GeLU remains the default in many contemporary large-scale Transformer implementations [102].

**Computational complexity analysis** The attention and feed-forward sub-layers are the most computationally intensive components of a Transformer, primarily due to extensive matrix multiplications. For a sequence length *n* and a model dimension *d*_model_, the scaled dot-product attention requires $O\left(n^{2}\cdot d_{k}\right)$ operations for computing the score matrix *QK* ^*T*^, and another $O\left(n^{2}\cdot d_{v}\right)$ for multiplying the softmax-normalized scores by *V*.

In multi-head attention, each of the *h* heads independently computes these operations with dimensions *d*_*k*_ = *d*_*v*_ = *d*_model_/*h*. Thus, considering all heads collectively, the complexity simplifies to [94]:

(7) $$O\left(n^{2}\cdot d_{\text{model}}\right)$$

The feed-forward network involves two large matrix multiplications:

– an *n* × *d*_model_ matrix multiplied by a *d*_model_ × *d*_ff_ matrix
– an *n* × *d*_ff_ matrix multiplied by a *d*_ff_ × *d*_model_ matrix

These operations yield a complexity of *O*(*n* ⋅ *d*_model_ ⋅ *d*_ff_). Since typically *d*_ff_ is a fixed multiple of *d*_model_ (e.g., 4 *d*_model_), the feed-forward complexity reduces to [94]:

(8) $$O\left(n\cdot d_{\text{model}}^{2}\right)$$

Combining both the attention and FFN complexities, the total computational complexity per Transformer layer becomes:

(9) $$O\left(n^{2}\cdot d_{\text{model}}+n\cdot d_{\text{model}}^{2}\right)$$

Both complexity terms can significantly affect runtime. Specifically:

– For long sequences (*n* very large), the attention complexity *O*(*n* ^2^) typically dominates.
– For wide models (*d*_model_ very large) with smaller sequence lengths, the FFN complexity becomes dominant.

In practice, modern large language models have thousands of dimensions for *d*_model_, and sequence lengths may range from hundreds to thousands of tokens. Consequently, both complexity terms are significant. These computations ultimately reduce to large-scale matrix-matrix multiplications, fundamental linear algebra operations that are efficiently executed using optimized hardware routines such as GEMM kernels on GPUs and TPUs [94], [100].

### 5.4 Challenges of training LLMs and photonic solutions

As described above, LLMs, powered by self-attention mechanisms as well as Transformer architectures, and further enhanced by training strategies like RLHF, have exhibited impressive performance across a broad range of tasks, including natural language understanding, generation, reasoning, and tool use [104], [105]. However, these capabilities come at the cost of substantial computational and energy demands [106]. The large model sizes, coupled with the intensive computation requirements of LLMs for both training and inference, necessitate deployment on high-performance hardware platforms. Moreover, the reliance on dense activation patterns and static computation reduces responsiveness and limits efficiency in real-time applications. Collectively, these challenges constrain the deployment of LLMs in energy-limited or latency-sensitive environments.

To address these limitations, a growing body of research has focused on improving the efficiency of LLMs through various model compression and optimization techniques. Quantization, for instance, reduces the precision of model parameters and activations, thereby decreasing memory usage and computational overhead with minimal performance degradation [107], [108], [109]. Other approaches include pruning, which removes redundant weights or attention heads [110], [111]; knowledge distillation, which transfers knowledge from large models to smaller ones [112], [113]; and the design of lightweight or sparsity-aware architectures that maintain performance while lowering resource consumption [114], [115], [116]. While these methods have achieved promising results, challenges remain in meeting the stringent efficiency requirements of real-time, low-power, or resource-constrained environments, especially for on-device or edge-level deployment [106], [117]. Therefore, current computing paradigms face severe bottlenecks and photonic computing has the potential to revolutionize LLM training and deployment by offering immense benefits of parallelism [42], in-memory computing [118] and energy efficiency [40]. These inherent advantages of photonic computing make it naturally superior for mathematical operations like matrix-vector multiplication, multiply-add accumulate, and dot products, as laid out in Section 5.3 above. What’s more, optical nonlinearities [71] can also efficiently enable standard activation functions in neural nets. Section 7.4 below touches upon some recent implementations of optical transformer accelerators [119], [120], [121] realized by photonic components. Nonetheless, there are areas where photonic computing lack convincing solutions, such as computing layer normalization, singular-value decomposition, and eigenvalue-eigenvector-related tasks, all of which are common in machine learning algorithms. Photonic computing also needs to demonstrate convincing computational complexity advantages over electronic computing, especially for ultra-large models and ultra-long sequences, for its full-scale adoption in the AI hardware domain. Besides, Sections 7.1–7.3 lists some additional challenges associated with mapping LLMs to photonic chips. Once these limitations are overcome, complex tasks requiring numerous electronic hardware to support computation will instead be more efficiently supported by new model architectures and efficient hardware solutions via photonics and optics.

## 6 Current challenges and future directions

There are challenges associated with photonics for next-gen AI computing, as well as future research directions being pursued by researchers.

### 6.1 Memory issue with long context window and long token sequences

Memory and context window: Photonic accelerators generally lack large on-chip memory to buffer long token sequences. Modern LLM inference may involve tens of thousands of tokens, requiring storage of activations, keys/values and intermediate states over the entire context. Without extensive SRAM or NVM on chip, photonic systems must stream these data in and out, reintroducing the von Neumann bottleneck. As Ning et al. observe, “data movement frequently constitutes the bottleneck of the entire system” – a problem that applies “not only in traditional electronic processors but also in optical processors”. In practice, limited on-chip memory forces a photonic LLM implementation to fetch context from external DRAM or disks, incurring latency and breaking the all-optical pipeline. Even as Feldmann et al. [42] proposed the well-known parallelized photonic in-memory computing using phase-change-material memory arrays, their memory capacity are constrained to 9 × 4 matrices, much smaller than modern LLM sizes. Emerging use cases like retrieval-augmented generation exacerbate this: performing near-real-time search and tokenization of multi-terabyte text corpora adds another round of expensive memory access. In short, the finite storage capacity of photonic chips constrains the feasible context length and throughput for LLMs, making long-sequence inference a major challenge.

### 6.2 Storage issue with mega-sized datasets on photonic computing systems

Storage and I/O bottlenecks: LLMs and their training or knowledge bases involve enormous datasets (multiple terabytes). Photonic accelerators still depend on high-bandwidth external memory and storage to feed these data. The I/O bandwidth needed can easily outstrip the available interfaces: even if the optical core is extremely fast, it is wasted if data cannot be streamed in quickly enough. Analysts warn of a growing “memory wall” for LLMs, where moving data becomes the dominant limitation. This is compounded by real-world workloads: for example, retrieval-augmented LLMs must repeatedly fetch and process large text blocks, placing severe demands on I/O. Some proposals (like co-locating non-volatile weight storage) can cut I/O (one study reports a 1,000 × reduction in chip I/O by using on-chip flash for weights), but even so the scale of multi-terabyte corpora means that data staging, caching, and bus bandwidth will remain critical bottlenecks in photonic LLM systems. Fortunately, there’s been works that strive to resolve this issue, such as in [122], where the authors proposed TeraPHY: a chiplet technology that enables low-power, high-Bandwidth (10s of Tb/s) in-package optical I/O. They demonstrated the power of TeraPHY by integrating it into the Intel Stratix10 FPGA multichip package for eifficient data transmission.

### 6.3 Precision and conversion overhead

Photonic computing is intrinsically analog, so representing high-precision tensors (needed for LLM inference) is difficult. State-of-the-art photonic Transformer designs rely on high-resolution ADCs/DACs to preserve accuracy, and these converters consume the majority of chip area and power. In some photonic transformer accelerators, the ADC/DAC circuitry occupied over 50 % of the chip and became a performance bottleneck. Reducing quantization error without blowing up conversion overhead is an ongoing challenge: low-bit converters or shared ADC schemes can improve area/energy, but may hurt model fidelity. Thus, finding optimal analog quantization schemes or mixed-signal architectures (perhaps using digital correction for a small fraction of values as in) is critical for next-generation photonic LLM chips. There has been recent works trying to resolve this challenge, such as in [123], where the authors proposed a quantization-aware training method that gradually performs bit reduction to layers in a mixed-precision manner, enabling them to operate lower-precision PNNs during deployment and further increase the computational rate of the developed photonic accelerators while keeping the energy consumption low.

### 6.4 Photonic attention architectures

A major research thrust is to implement transformer self-attention directly in optics. This involves designing tunable photonic weight elements and reconfigurable interferometer networks to compute QxK and V-weighted sums optically. For example, photonic tensor cores are being developed that use Mach–Zehnder interferometer (MZI) meshes or other crossbar arrays to carry out large matrix multiplications in parallel [119]. Tunable weights may be realized by phase shifters, microring modulators, or even magneto-optic memory cells: one recent proposal used Ce:YIG resonators to store multibit weights, enabling non-volatile, on-chip optical weight storage [120]. In addition, delay-based schemes from reservoir computing could provide temporal context: long optical delay lines or series-coupled microrings have demonstrated very high memory capacity for sequential tasks [121]. A promising vision is an all-optical transformer block where dynamic weight matrices are programmed into an optical mesh and past token states are held in transit delays, allowing the self-attention kernel to be evaluated at light speed. Recent designs like lightening-transformer [119] (a “dynamically-operated photonic tensor core”) and HyAtten validate this approach: they achieve highly parallel, full-range matrix operations while minimizing off-chip conversion. Continued work on integrated optical buffers, high-bandwidth modulators, and photonic softmax approximations will advance this direction.

### 6.5 Neuromorphic and spiking photonic LLMs

Another pathway is to recast LLM inference in a neuromorphic, event-driven paradigm. Spiking neural networks (SNNs), as discussed in Chapter 2 and 4, process data as sparse asynchronous events, which naturally match photonics’ strengths. Indeed, all-optical spiking neural networks have been demonstrated on chip using phase-change neurons and laser pulses. One could imagine encoding a token stream as optical spikes or pulses and using a photonic SNN with synaptic weights to perform sequence processing. Hybrid photonic–spintronic designs [87] could play a role here: spintronic devices (magnetic tunnel junctions, phase-change synapses) provide compact non-volatile weight storage and can interface with optical neurons. Recent work on photonic in-memory weights (using magneto-optics) [120] and on photonic neuromorphic accelerators leveraging extreme sparsity [124] suggests that embedding non-linear, event-driven components on a photonic chip is feasible. Such architectures could exploit data sparsity (most tokens only weakly excite the network) and update weights only when events occur, greatly reducing energy. Exploring spiking attention models or sparse transformer variants on photonic neuromorphic hardware is an exciting future direction for low-power LLM inference.

### 6.6 System integration and Co-design

Finally, scaling LLMs on photonics will require co-design across layers. This includes integrating photonic processors with advanced optical I/O and memory hierarchies, as well as co-optimizing algorithms for the hardware’s strengths. For example, recent fully integrated photonic DNN chips (fabricated in commercial foundries) show it is possible to perform all neural network computations optically on-chip [125]. Extending such integration to transformer-scale models will demand dense wavelength-division multiplexing, optical network-on-chip fabrics [126], and novel packaging (e.g. co-packaged optics) to boost throughput. Meanwhile, software tooling (quantization, parallelism, placement) must adapt to photonic hardware. Efforts on photonic-electronic co-packaging [127] and compute-in-memory architectures offer a roadmap: by tightly coupling photonic tensor cores with co-located memory banks and control logic, one can mitigate the von Neumann overhead. In the longer term, success will likely come from global co-design – matching transformer algorithms (sparsity, low precision, model partitioning) to the capabilities of non-von Neumann photonic chips. These combined hardware/software innovations could unlock the massive parallelism of light for next-generation LLM workloads.

## 7 Conclusions

In conclusion, developing and integrating diverse, powerful photonic and spintronic devices and circuits has significantly broadened the horizons for reshaping computational AI paradigms. As introduced in the initial chapters, MRRs have enabled WDM, thereby substantially increasing data density and scalability of optical neural network systems, while simultaneously introducing critical nonlinear effects necessary for practical neural network functionality. Additionally, by controlling the phase difference in the optical paths through opto-electronic or thermo-optic effects, MZIs can help implement programmable weight matrices essential to neural network computation. Furthermore, the novel design and usage of the metamaterials have enabled even more applications, introducing linear and nonlinear computations through diffraction and interference of the light. Additionally, the photonic devices incorporating 2D materials such as graphene and TMDCs, alongside innovative spintronic neuromorphic devices, are actively being studied, showing promising results in the optical neural networks.

In the meantime, the rapid advancement of LLMs and transformers has driven remarkable progress in machine learning. The current popular models are based on a transformer architecture through multi-head self-attention, feed-forward neural networks, and advanced positional encoding methods, significantly enhancing parallel computational efficiency. Innovations such as CoT prompting, self-reflection techniques for reasoning, RLHF, Toolformer integration, and memory management solutions such as ALiBi have facilitated the development and deployment of sophisticated commercial models like ChatGPT, LLaMA, and DeepSeek, demonstrating remarkable performance across multiple scenarios and applications. On the other hand, complex tasks require numerous electronic hardware to support computation, which shows an urgent need for new model architectures and efficient hardware solutions such as photonics.

That being said, difficulties and challenges remain, such as on-chip memory capacity and data storage capabilities, which continue to constrain the handling of extensive datasets and long context windows characteristic of modern LLMs. Additionally, precision limitations and the overhead associated with analog-to-digital conversions, effective integration of nonlinear operations directly onto photonic chips, and seamless system-level integration persist as obstacles. With the constant study in the field of photonics and spintronic neuromorphic devices, more viable solutions to these challenges will eventually arise. Future research must prioritize scalability, material stability, refined integration methodologies, and the continued evolution of neuromorphic and SNN frameworks to exploit the potential of photonic computing fully.

Eventually, research efforts integrating photonics, 2D materials, spintronics, and advanced neural architectures will catalyze transformative progress, paving the way toward energy-efficient, scalable, and high-performance computing infrastructures. These innovations will support increasingly complex and demanding AI tasks and applications, and bring LLMs into a new era.
